# Invariant HVC size in female canaries singing under testosterone: Unlocking function through neural differentiation, not growth

**DOI:** 10.1073/pnas.2426847122

**Published:** 2025-10-20

**Authors:** Shouwen Ma, Carolina Frankl-Vilches, Manfred Gahr

**Affiliations:** ^a^Department of Behavioural Neurobiology, Max Planck Institute for Biological Intelligence, Seewiesen 82319, Germany

**Keywords:** songbird, brain, plasticity, testosterone, in-vivo imaging

## Abstract

Testosterone administration to nonsinging adult female canaries induces song, making this a model for behavioral plasticity and its underlying neural mechanisms in vertebrates. The song control nucleus HVC is traditionally believed to undergo a substantial size change when transitioning from a nonfunctional to a functional (song-producing) state. Using 2-photon in vivo imaging, we tracked the spatial distribution and anatomical properties of HVC neurons over several weeks of testosterone-induced song development. Surprisingly, despite ultrastructural changes of HVC neurons, testosterone did neither alter neuronal spacing nor HVC size. Instead, spatial transcriptomics revealed that testosterone modulates gene networks throughout HVC, aligning transcriptomic profiles between its peripheral and central HVC regions in singing birds, thereby mimicking the histological appearance of an enlarging HVC. Our results demonstrate that changes in HVC size in adults reflect phenotypic changes in neurons within a stable framework. Importantly, the nonfunctional state is not associated with a reduced brain area volume, preserving HVC’s capacity for functional differentiation throughout life.

## Significance

In many songbird species, singing can be induced in otherwise nonsinging females or is expressed only during certain life stages. These transient behaviors have been attributed to testosterone-driven growth and regression of song control brain regions. We show that in adult female canaries, the size of these regions remains constant; instead, testosterone alters the phenotype and activity of their component neurons. Thus, the adult brain retains the capacity to respond to testosterone, allowing behaviors such as song to reemerge even after years of silence.

Large-scale plasticity in adult human brains is well documented, particularly following injuries such as strokes or limb loss (1). Comparable large-scale plasticity is observed in many vertebrate species related to their seasonal lifestyle (2–7). A common assumption is that absence or loss of function correlates with reductions in neural structure, such as neuron numbers or brain volume. Notable examples are the lack of singing in adult female songbirds and the seasonal singing behavior of songbirds, with singing production prominent during certain stages of life such as the breeding seasons and reduced during nonbreeding periods in males (8). Functional MRI studies have demonstrated marked seasonal changes in the parcellation of the adult songbird brain, including the song control system and sensory circuits such as the visual system (9–12). These changes are strongly influenced by testosterone, particularly evident in male canaries (*Serinus canaria*) (13–15) and replicable in female canaries by testosterone treatment (3, 16–19). Such treated females transiently evince the capacity for male-like songs (14, 16, 18–20), showcasing extensive neural changes in the song control circuit, notably in the size of brain areas responsible for song control (3, 14, 16–18). These structural alterations correlate with massive shifts in gene expression within the HVC (used as proper name) (21, 22), a critical nucleus in the songbird brain’s song control circuit, underscoring its pivotal role in the plastic ability to sing or not to sing.

**Fig. 1.**
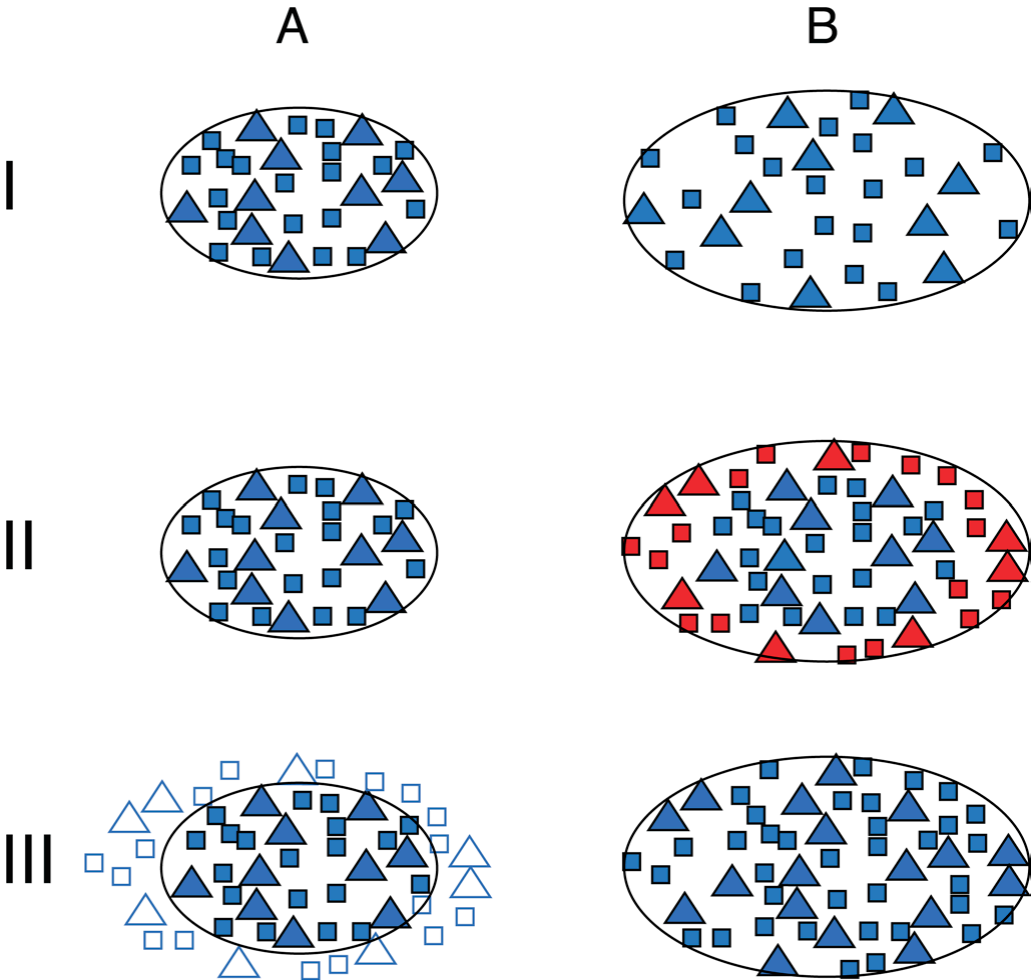
Models (I to III) illustrating possible mechanisms to explain the apparent increase in HVC size associated with the transition of testosterone levels from low (*A*, mimicking the nonbreeding season) to high (*B*, mimicking the breeding season), reflecting seasonal transitions. Black lines indicate the borders of the HVC, delineated based on the cytoarchitecture of fully differentiated neurons (filled symbols). Squares and triangles depict two neuron types, with opened squares and triangles being undifferentiated and closed squares and triangles being fully differentiated neurons. Model I. Expansion by spacing out of existing neurons; Model II. Addition of newly born neurons (red squares and triangles) to the margins of the HVC; III. Differentiation of existing neurons at the borders of the HVC during the transition from low to high testosterone state.

**Fig. 2.**
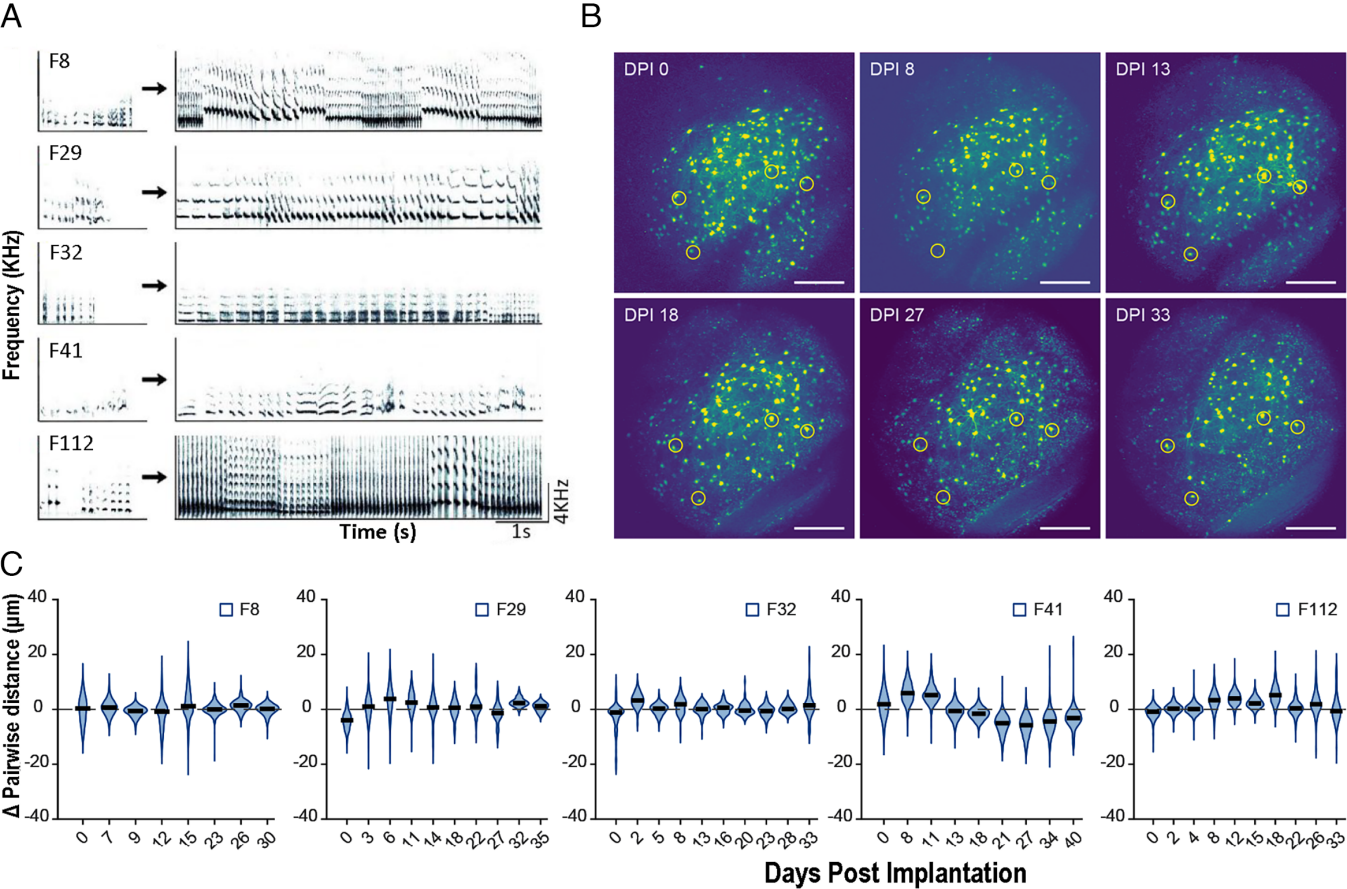
Song development and HVC size stability during testosterone-induced singing in adult female canaries. (*A*) Sonograms of the developing songs of five adult female canaries (F8, F29, F32, F41, and F112) from first highly variable subsongs (recorded at 5 d postimplantation of testosterone [DPI], left column) to well-developed so-called full songs achieved by all females at the latest on 33 DPI (right column). (*B*) Maximum intensity projections from time-lapsed in vivo 2-photon imaging of retrogradely labeled HVC_X_ neurons in one testosterone-treated female (F41) are shown at various time points: DPI 0, 8, 13, 18, 27, and 33 (transverse planes of HVC). Yellow circles mark four example neurons at different DPIs. Neurons were retrogradely labeled with the viral vector scAAV-DJ/9-CMV-eGFP (eGFP). Background variations in these maximum projection images reflect differences in maximum pixel intensities from dorsal to ventral planes on different days. Slight changes in pairwise distances between observation days are likely due to measurement errors related to the optical resolution limits (*Materials and Methods*). (Scale bar, 200 µm.) (*C*) The HVC size remains stable when delineated by the distribution of HVC_X_ neurons. The changes in pairwise distances between eGFP-labeled HVC_X_ neurons across consecutive days of testosterone treatment in five females (F8, F29, F32, F41, and F112) were minimal, averaging 0.3 ± 4.7 µm. In the violin plot, outlines were determined by the kernel density estimation of the probability density function. Solid black lines indicate medians, while the dashed gray line indicates the zero-reference, corresponding to no change in pairwise distances between neurons. For detailed statistical results, see *SI Appendix*, Table S2: the observed cell shifts of all females and all observation days differed significantly from the 10%-expansion model (*SI Appendix*, Table S2). For 5% expansion models, 40 of 47 observation days differed significantly; and even for 3%-expansion, 32 of 47 observation days differed from the predictions (one-way ANOVAs with f(1,93) = 73.5, *P* < 0.001, followed by Tukey post hoc tests).

**Fig. 3.**
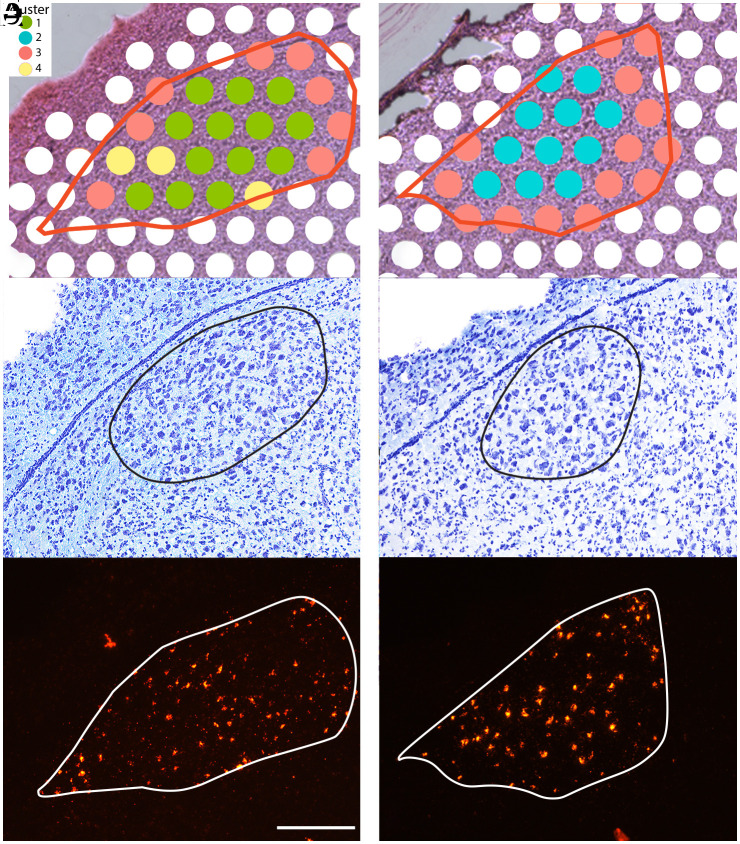
The size of molecular-defined (*A* and *D*) and cytoarchitectonically defined (*B* and *E*) HVCs increases within the retrogradely defined (*C* and *F*) limits of the HVC in testosterone-treated females (*A*–*C*) compared to placebo-treated females (*D*–*F*). The molecularly defined HVC subareas (*A* and *D*) result from the clustering of spatial transcriptomes (*SI Appendix*, Fig. S5) located within the retrogradely defined HVCs (red lines in *A* and *D* that correspond to white lines in *C* and *F*). Each 55-µm spot in (*A*) and (*D*) represents one spatial transcriptome. The ventricle dorsal to HVC separates the caudal forebrain from the hippocampus. Cluster 1 spots (green spots; *A*) were located in the center of HVCs in testosterone-treated females only, and Cluster 2 (blue spots; *D*) were in the center of HVCs of placebo-treated females only. Cluster 3 (red spots, *A* and *D*) are spots mainly located at the margins of retrogradely defined HVCs and are reduced in number in testosterone-treated HVCs (*A*). Cluster 4 (yellow spots; *A*) likely represents a mixture of cells that otherwise form Clusters 1 and 3. The outlines of the cytoarchitectonically defined (Nissl-staining) HVCs (*B* and *E*) are smaller compared to the retrograde-defined HVC (*C* and *F*) in the placebo-treated females (*E* and *F*). The cytoarchitectonically defined HVCs coincide well with Cluster 1 (*A*) in testosterone-treated females and Cluster 2 (*B*) in placebo-treated females. The larger red objects in the upper part of (*C*) and lower edge of (*F*) are artifacts. The sections shown in (*A*) and (*D*) are adjacent to the section shown in (*B)* and (*C*) and in (*E*) and (*F*), respectively. This leads to minor differences in the outlines of sections (*A*–*C*) and (*D*–*F*), respectively. (Scale bar, 160 µm.)

The HVC is essential for song sequential patterning (23, 24). Changes in brain segmentation, specifically alterations in HVC size, might be due to variations in neuron and glia cell numbers, as well as to modifications in the vascular compartment within the HVC (25–27). Alternatively or in addition, such changes could result from reorganization at multiple structural levels within existing neurons, such as size, cell nucleus size, perineuronal nets, dendritic arborization, and overall RNA and protein content of cells (28) due to transient changes in gene networks. These changes may enhance the detection of HVC boundaries without altering the overall HVC size, as has been suggested, albeit controversially, for the male canary HVC (29, 30). Therefore, we propose three hypothetical models for HVC volume increase (Fig. 1): Model I, expansion of the HVC via increased spacing among existing neurons; Model II, addition of new neurons at the HVC margins; and Model III, differentiation of existing neurons at the borders of the currently detectable HVC. Determining the level of organization at which changes of brain segmentation occur is crucial for identifying the underlying mechanisms and gaining a comprehensive understanding of adult brain plasticity. To address this, we tracked the testosterone-induced changes in the adult female HVC using in vivo 2-photon imaging of prelabeled neurons throughout song development over several weeks (Fig. 2). Remarkably, this approach revealed that HVC volume remained constant. To investigate the discrepancy between the observed invariant HVC volume in our imaging and the increase in HVC size reported in traditional histology after testosterone treatment (*SI Appendix*, Fig. S1*B*), we performed spatial transcriptomics of HVCs from testosterone- and placebo-treated birds (Fig. 3). This comparison showed that testosterone harmonizes the gene networks and the associated biological processes in large parts of the HVC, thereby simulating HVC growth within its fixed boundaries, indicating that the segmentation of the adult brain remains stable. In its nonfunctional state, the HVC neuron populations of females remained intact, enabling the HVC to respond to elevated testosterone throughout life (*SI Appendix*, Fig. S10).

## Results and Discussion

### Changes in Song and HVC, Visualized Using Nissl Staining, in Testosterone-Treated Canaries.

Testosterone treatment significantly increased the hormone levels in all treated females compared to their pretreatment baseline, while levels in placebo-treated females remained consistently low (*SI Appendix*, Fig. S1*A* for statistics). Testosterone treatment induced the onset of song development within a few days (5 ± 2 d). As song development progressed, syllables became more differentiated in their spectro-temporal structure, leading to distinct syllables repeated in sequences at 28 ± 5 d (Fig. 2*A*). The organizational pattern of songs produced by testosterone-treated females closely resembled male songs during their reproductive season, when natural testosterone levels are elevated. However, as reported previously (16, 18, 19), female songs included fewer syllable types in (4 ± 3 syllables) than male songs. As anticipated, Nissl-stained sections of HVC—a standard cytoarchitectonic method to quantify the volume of brain areas—revealed significantly larger HVC volumes in testosterone-treated females (f(8) = 4.69, *P* = 0.0008, one-sided *t* test) 5 to 6 wk posttreatment onset. The HVC volumes of testosterone-treated females averaged 0.206 ± 0.028 mm^3^ (mean ± SD, N = 6), compared to 0.110 ± 0.036 mm^3^ (mean ± SD, N = 4) in placebo-treated females (*SI Appendix*, Fig. S1*B*). In a parallel study, cytoarchitectonically defined HVC volumes showed significant increases as early as 14 d following testosterone treatment (21). Based on these phenotypic changes, we expected to observe an increase in HVC size during testosterone treatment in our 2-photon imaging data.

### HVC Neuron Distribution Is Unchanged with 2-Photon Imaging During Testosterone-Driven Song Development.

The HVC contains two densely intermingled types of projection neurons: HVC neurons projecting to the basal ganglia Area X (HVC_X_) and HVC neurons projecting to the robust nucleus of the arcopallium (RA) (HVC_RA_) (31). Typical cell clusters within HVC consist of these two neuron types alongside interneurons (31, 32).

Among the three models proposed to explain changes in HVC volume (Fig. 1), Model II (addition of new neurons at the HVC margins) can be excluded because HVC_X_ and HVC_RA_ neurons are consistently adjacent throughout HVC (31), and given that HVC_X_ neurons are not born in adult brains (29, 33), Model II would predict an HVC rim devoid of HVC_X_ neurons in testosterone-treated females at advanced stages of song development—a feature that was never observed (31, 32).

To determine whether Model I (increased spacing of existing neurons) or Model III (differentiation of existing neurons at the borders of the currently detectable HVC) explains the testosterone-driven increase in HVC volume during song acquisition in female canaries (Fig. 1), we analyzed the spatial distribution of HVC_X_ neurons throughout song development. Prior to testosterone treatment, HVC_X_ neurons were retrogradely labeled by injecting scAAV-DJ/9-CMV-eGFP (34) into Area X. We analyzed the anatomical position of HVC_X_ neurons numbered 275, 190, 44, 193, and 133 of five female canaries, labeled F8, F29, F32, F41, and F112, respectively. Using in vivo 2-photon microscopy, we tracked the precise anatomical positions of each labeled HVC_X_ neuron throughout testosterone-induced song development, exemplified by female F41 (Fig. 2*B*) and results summarized for all females in Fig. 2*C*. Time-lapsed in vivo 2-photon imaging showed no significant positional shifts in HVC_X_ neurons during song development (mean slope: 0.01, R^2^ < 0.05, mean cell shift: 0.3 ± 4.7 µm, N = 5 birds, Fig. 2*C*). Assuming a HVC volume of 0.125 mm^3^ corresponding to a cube side length of 500 µm, mathematical models predict that HVC expansions of 5%, 20%, 50%, and 90% would require average cell position shifts of 4 ± 1 µm, 15 ± 4 µm, 35 ± 10 µm and 55 ± 17 µm (means ± SD), respectively (*SI Appendix*, Fig. S2). In contrast, we observed only 0.3 ± 4.7 μm of mean shifts (Fig. 2). Across all females and time points (Fig. 2), all observed data differed significantly from the 10%-expansion model (*SI Appendix*, Table S2). For the 5%-expansion models, 85% (40 of 47) observation days were significantly different from the predictions and even for 3%-expansion models, 68% (32 of 47) observation days still differed from the predictions (for all comparisons: one-way ANOVAs with f(1,93) = 73.5, *P* < 0.001, followed by Tukey post hoc tests; *SI Appendix*, Table S2). Further, the majority of similarities between observation days and 5% or 3% expansion models reflected decreases in pairwise differences, indicating shrinkage (*SI Appendix*, Table S2). The remaining similarities to the 0%-, 2%-, 3%-, and 5%-expansion models are likely due to technical shortcomings such as limited optical resolution (*Materials and Methods*). Given that our 2-photon imaging readily resolves changes in the spines and boutons (*SI Appendix*, Fig. S9), the expected cell shifts cannot have been overlooked. Similarly, GFP-labeled migrating neurons can be detected in the brains of adult birds using 2-photon imaging (35). Further, if HVC volume increased as in juvenile male zebra finches, the spacing of retrogradely traced HVC_X_ neurons increased (36). Therefore, the absence of these positional shifts effectively excludes Model I (Fig. 1), which predicted an increase in distance between neighboring neurons as the explanation for HVC growth in the adult canaries.

To further support our findings, we expanded our 2-photon analysis to include all HVC neurons. Given that our 2-photon imaging readily resolves changes in the spines and boutons (*SI Appendix*, Fig. S9), the expected cell shifts cannot have been overlooked. Similarly, GFP-labeled migrating neurons can be detected in the brains of adult birds using 2-photon imaging (35). Further, if HVC volume increased as in juvenile male zebra finches, the spacing of retrogradely traced HVC_X_ neurons increased (36). Prior to testosterone treatment, we injected AAV9-CAG-GCaMP6s into the HVC of one female canary, randomly labeling neurons including HVC_X_ cells and GABAergic interneurons (*SI Appendix*, Fig. S3). Consistent with our observations for HVC_X_ neurons (described above), the distances between these various neuron types remained stable during the entire testosterone-driven song development (*SI Appendix*, Fig. S4). All measurements differed significantly from the predictions of the 5%- and 10%-expansion models (one-way ANOVAs with F(1, 25) = 86.6, *P* < 0.001, followed by Tukey post hoc tests, *SI Appendix*, Table S2). These results suggest that the spatial arrangement of most HVC neurons is maintained during song development of adult canaries.

### If Neuron Spacing Is Invariant, What Then Drives the Apparent Increase in Volume of the HVC Observed in Cytoarchitectonic Analyses (3, 14, 16–18, 22) During Testosterone Treatment (testing the Modell III)?

The constant spatial distribution of HVC_X_ neurons throughout song development aligns with the prediction of Model III, which suggests that the differentiation of existing neurons at the margins of the HVC could explain the observed volume increase. To understand this, we compared gene expression in the HVC of testosterone-treated females (N = 4) and time-matched placebo-treated females (N = 4) using spatial transcriptomics, which provides spatial insight into transcriptomic differences in the HVCs. Spatial transcriptomics provides a combined transcriptome of all cells in an intact tissue patch with a diameter of 110 µm, corresponding to an average of 100 cells in DAPI-stained HVC sections, and thus provides information on transcriptional heterogeneity within a small HVC cell population, while the contribution of different cell types to this heterogeneity remains unclear (37). Testosterone acts on cells via two transcription factors, the androgen receptor (AR) and after aromatization into estrogens, the estrogen receptor. In HVC, ARs are expressed primarily in both types of projection neurons (38, 39), while estrogen receptors are mainly expressed in X-projecting HVC neurons (29). In particular, we compared the molecularly defined HVC (spatial transcriptomics) with the HVC delineated by connectivity (retrograde tracing) and by cytoarchitecture (Nissl staining) (Fig. 3 and *SI Appendix*, Fig. S5). In spatial transcriptomics, forebrain sections containing HVC are subdivided into 55 µm diameter spots (*Materials and Methods*), each representing a unique transcriptome. For further bioinformatic analysis, we considered the transcriptomes from all spots in which more than 50% of the area fell within the retrogradely defined HVC boundaries, combining a total of 142 transcriptomes of testosterone- (75 transcriptomes) and placebo-treated (67 transcriptomes) females (*SI Appendix*, Fig. S5). This combined analysis of testosterone-treated and placebo-treated birds revealed only four clusters (*SI Appendix*, Fig. S5). When mapped back onto HVC, Cluster “1” (green spots) aligned with the cytoarchitectonically defined HVCs in only testosterone-treated birds, while Cluster “2” (blue spots) coincided with the cytoarchitectonically defined HVCs of only placebo birds; spots of Cluster “3” (red) were located at the edges of the cytoarchitectonically defined HVCs, and the sparse spots of Cluster “4” (yellow) were scattered, but mainly at the edges. The yellow transcriptomes (Cluster 4) are probably the results of a mixture of cells otherwise forming transcriptomes of Clusters 1 and 3 or 2 and 3, respectively, and are not further considered due to their low number (Fig. 3 and *SI Appendix*, Fig. S5). The ratios of molecularly defined HVC area to Nissl-defined HVC area were similar between placebo-treated and testosterone-treated females (t(4) = 0.81, *P* = 0.24, one-sided *t* test). However, the ratios of molecular-defined to Nissl-defined HVC areas (ST/Nissl) differed from the ratios of molecular-defined to retrograde-defined HVC areas (ST/Trace) in placebo-treated females (t(4) = 7.23, *P* = 0.001, one-sided *t* test) but not in testosterone-treated females (t(4) = 1.55, *P* = 0.1, one-sided *t* test) females (*SI Appendix*, Table S1). Thus, the cytoarchitectonically defined HVC size correlated with the areas of Cluster 1 and Cluster 2, respectively, while the area of Cluster 1 (testosterone-treated females) was significantly larger than that of Cluster 2 (placebo-treated females), all within the invariant boundaries of retrogradely defined HVCs (Fig. 3).

A comparison of differentially expressed genes (DEGs) between the transcriptomes of Cluster1, Cluster 2, and Cluster 3 revealed larger differences between Clusters 1 and 3 (711 DEG with *P* < 0.01) than between Clusters 2 and 3 (391 DEG with *P* < 0.01) and between Clusters 1 and 2 (412 DEG with *P* < 0.01) (see *SI Appendix*, Tables S3–S5 for most significant DEGs). A gene ontology analysis of all DEGs of the transcriptomes of Clusters 1, 2, and 3 showed that Clusters 1 and 2 differ from Cluster 3 mainly in biological processes related to metabolism and nervous system development, in particular those involving synapse and axon-related processes (*SI Appendix*, Fig. S8). Comparisons between Clusters 1 and 2 revealed that their biological processes differ mainly in terms of metabolism (*SI Appendix*, Table S5 and Fig. S8). The gene ontology differences between Clusters 1 and 3 and Clusters 2 and 3 were more pronounced than those of Clusters 1 and 2. This pattern is consistent with the observation that the transcriptomes of Clusters 1 and 2 were located in the central HVC, with similar cytoarchitectural phenotypes in both testosterone- and placebo-treated females, whereas the transcriptomes of Cluster 3 originate from the HVC margins, which differ in their cytoarchitecture from the central HVC of both treatment groups (Fig. 3). The gene expression changed even in the cytoarchitectonically defined HVC part of nonsinging females after testosterone treatment (*SI Appendix*, Table S5), suggesting that the entire HVC adapts at the molecular level to support the singing function. In particular, the mRNA of estrogen receptor alpha (ESR1) of Cluster 1 is upregulated as compared to Clusters 2 and 3, the AR and 5α-reductase (SRD5A2) is upregulated in the transcriptomes of Cluster 1 and Cluster 2 compared to Cluster 3; 5α-reductase converts testosterone into the more potent androgen 5α-dihydrotestosterone. One example of a gene upregulated in transcriptomes of Cluster 3 compared to those of Clusters 1 and 2, is cholecystokinin (CCK). The differential expression of these genes in HVC center versus HVC margin was verified in RNAscope in situ hybridizations as depicted in *SI Appendix*, Figs. S6 and S7. These differently expressed genes suggest that gene regulation in the transcriptomes of Cluster 1 reacts more sensitively to testosterone than in Clusters 2 and 3 and that Cluster 2 in turn is more testosterone-sensitive than Cluster 3. Since histological staining techniques like Nissl staining primarily label ribosomal RNAs (40), the high cellular activity under the influence of testosterone manifests itself in an apparently enlarged HVC in Nissl-stained sections (Fig. 3). Likewise, metabolic cellular changes might facilitate the detection of HVC in in vivo imaging techniques such as fMRI (9–12), potentially reflecting an increased functional HVC.

Regarding gene ontology terms related to neural functions, the increased overt HVC size coincides with gene expression related to the axon and synapse functions. To investigate this further, we analyzed the morphology of HVC_X_ neurons during testosterone-driven song development. The soma size of HVC_X_ neurons did not show significant changes during song development; the mean soma size was 13.4 ± 2.0 µm^2^ at start of treatment (days postimplantation, DPI 0) and 13.1 ± 1.8 µm^2^ at the full song stage (DPI 28 to DPI 42; two-way ANOVA, F = 0.04, *P* = 0.85; N = 605 cells in 5 birds, *SI Appendix*, Fig. S9*A*). While the density of boutons of HVC neurons remained consistent during early song development (*SI Appendix*, Fig. S9*C*), boutons showed dynamic changes such as growth, retraction, and remodeling (indicated by magenta arrows in *SI Appendix*, Fig. S9*D*) and an overall increase in density in later stages of song development (*SI Appendix*, Fig. S9*E*). The bouton density of HVC_X_ neurons doubled, from ca. 0.1 boutons per µm axon during early song onset (DPI 1 to DPI 10) to ca 0.2. boutons per µm axon in the full song phase (DPI 30 to DPI 42) (*SI Appendix*, Fig. S9*E*, R^2^ = 0.33; two-way ANOVA, F = 18.07, *P* < 0.001; N = 16 branches of 3 birds). Further, our 2-photon imaging captured the transformation of HVC synapses from hair-like structures to mushroom-shaped heads (*SI Appendix*, Fig. S9*D*). These results show ultrastructural dynamics in these otherwise stable HVC neurons. Although many processes are changed by testosterone, the combined modifications in neuronal ultrastructure (synapses, axons) and metabolism (mitochondria) might be of particular importance for the “singing” HVC. In mice, for instance, the emergence of mitochondria at the base of spines seems to be important for synaptic plasticity (41).

We show that HVC_X_ neurons, and likely most other HVC neurons, remain stable components within the HVC in adult testosterone-induced singing female canaries. These cells maintain their spatial distribution in the caudal forebrain independently of hormonal fluctuations. However, their ultrastructure, such as bouton density, undergoes changes during song development, which is related to important transcriptional changes within HVC subareas induced by testosterone. These results support Model III, explaining the apparent HVC growth as local differentiation, i.e., as the change in phenotypes of existing neurons and other cell types within an invariant scaffold of HVC neurons, spanned out by HVC_X_ neurons and possibly other HVC cell types. Thus, the previously reported HVC enlargement in testosterone-induced singing female canaries (13, 14, 16–18, 22) represents a phenotypic hormone-induced morphological change of the cells within HVC compartments, rather than the volumetric expansion. In agreement, depletion of neural progenitors—and thus of newly recruited HVC neurons—does not affect the testosterone-induced increase in HVC volume (as defined by Nissl-stains) in adult female canaries (17). In relation to the current results, it has been suggested that the seasonal growth of HVC in male canaries represents a delineation result rather than a real volume change (29, 30, 42). This proposal for male canaries was based on comparative anatomical techniques rather than 2-photon imaging used in the current study. In male canaries, seasonal song changes are accompanied by substantial changes in the HVC transcriptomes (21) that are quantitatively comparable in scale to those observed in testosterone-treated females shown here, providing a molecular explanation for changes in male HVC cytoarchitecture. Therefore, changes in HVC size observed with Nissl-staining reflect alterations in functional HVC size (3, 13–19) rather than a change in HVC size based on the altered spatial distribution of its component cells.

The differentiation model proposed here for adult song development in canaries proposes a framework that may also apply to similar observations in reptiles, birds, and mammals (2, 4–6, 43–47). This model, in particular, explains how the overt size of brain areas in general can change drastically even within just a few days (2, 46, 47). Our model of “illusionary” growth through cellular differentiation does not rule out the possibility of recruitment of newborn neurons into the adult HVC (25, 48), but neither neurogenesis nor cell death is required. Thus, differentiation alone can explain many observations of sex-specific or transient changes in the size of brain areas in which neurogenesis does not occur in adult animals, such as the song control nucleus RA and the preoptic area in songbirds (3, 46–48) and most brain areas in mammalian species (49). While transient changes in brain region volume of females and males due to neuron death and recruitment are theoretically possible, they cannot be derived from cytoarchitectonic staining alone and require case-specific verification. To date, the only well-documented such example remains the seasonal loss of interneurons in cortical layers in shrews (7). The differentiation, instead of growth, of the HVC following testosterone treatment of adult female canaries does not support the traditional “use it or lose it” paradigm for adult neuron populations, highlighting their retained functional potential throughout life. In relation, testosterone induced singing even in two 7-y-old female canaries (*SI Appendix*, Fig. S10), an age exceeding by far their natural lifespan in the wild. These findings suggest that brain segmentation and intrinsic functions of specific neuronal populations remain stable, even in the absence of regular use. In female songbirds, this could explain how females of many songbird species are occasionally able to sing as adults or only during specific life stages (8).

## Materials and Methods

### Animals.

Fourteen adult female canaries (*S. canaria*), aged between 300 and 600 d old, were obtained from our breeding colony in Seewiesen, Germany. Of these, ten females were implanted with testosterone: Six were used for 2-photon imaging and four for spatial transcriptomics. Five of the females used for 2-photon imaging were injected with an eGFP AAV virus into Area X while a further female was injected with a GCaMP AAV virus into HVC (see below). Four additional females were implanted with placebos and used for spatial transcriptomics. For spatial transcriptomics, the eight birds were injected with Retrobeads into Area X. Further, two 7-y-old female canaries were included to study testosterone-induced singing in very old birds. Throughout the experiments, the animals were kept in a 14 h:10 h light–dark cycle, with food and water available ad libitum. Handling of the animals and experimental procedures were conducted according to the ethical principles and guidelines for animal experiments in Germany and experimental protocols were approved by the government of Upper Bavaria (ROB-55.22532.Vet_02-18-142).

### Testosterone Treatment and Analysis.

The vocal behaviors of the naive adult female canaries were initially monitored for 1 wk to confirm the lack of song production in these birds, as rare cases of spontaneous singing may occur in female canaries (50). Subsequently, females were implanted with 8-mm long silastic tubes (Dow Corning) with an inner diameter of 1.47 mm, filled with crystalline testosterone (Sigma-Aldrich), or placebo-treated with an empty implant. Implants were inserted under the skin of the upper back following local disinfection with 70 % ethanol and local anesthesia using 2% lidocaine hydrochloride (Xylocaine gel). The day of testosterone or placebo implantation was defined as DPI 0 (day postimplantation 0).

Testosterone plasma concentrations were measured using radioimmunoassays following published methods (51). In brief, blood samples were taken from the brachial wing vein using heparinized capillary tubes. The blood plasma was gained by centrifugation at 2,500 rpm for 10 min and stored at −20 ^o^C until analysis. Testosterone was extracted from plasma using a modified version of the partial purification on diatomaceous earth/glycol column method. The recovery rate for testosterone was 0.83% ± 0.05% (mean ± SD). The lower detection limit of the standard curves was determined as the first value outside the 95% CI for the zero standard (B_max_). The detection limit for testosterone was 0.12 pg/mL plasma. All samples fell within the detectable range for testosterone.

### Surgical Procedure for Retrobeads Injections and Viral Vector Injections.

The self-complementary retrograde viral vector for eGFP (scAAV-DJ/9-CMV-eGFP) was acquired from the Viral Vector Facility of the University of Zurich and ETH Zurich (34). The viral vector for GCaMP (AAV9.CAG.GCaMP6s.WPRE.SV40) was acquired from Addgene (#100844). Due to the AAV9 serotype and the ubiquitous CAG promotor, the latter virus randomly infects cells around the injection site and gets active in many HVC cells. The canaries were given general analgesia 15 min before surgery by injection of metamizole (100 to 150 µg/g body weight) into the pectoral muscle. The birds were anesthetized with isoflurane inhalation (0.8 to 1.8 % at 0.5 L O_2_/min). After administration of the local anesthetic procaine (2%), a vertical incision was made in the skin over the skull and a craniotomy was performed. For injection of eGFP into Area X, craniotomy was at 1.4 mm lateral, 5.6 mm rostral, and 3.3 mm ventral with respect to the Y-sinus zero point, with an 80° angle between the ear bar and the beak; for the injection of GCaMP into the HVC craniotomy was at 2.4 mm lateral, 0 mm rostral, and 0.3 mm ventral with respect to the Y-sinus zero point, with a 45° angle between the ear bar and the beak. At each target site, 400 nL of viral vector (0.3 to 3 × 10^13^ vg/mL) was injected using glass pipettes attached to a manually operated hydraulic injector. Immediately after injection of viral vectors, a chronic cranial window with a headpiece was placed over the HVC. The procedure involved removing the skull above the HVC, leaving the dura intact for long-term imaging. A custom-cut cover glass was fitted into the cranial opening and secured to the skull with Kwik Sil (WPI) and UV-curing dental cement (Tetric evoflow). A custom-made titanium headpiece with a 7-mm diameter opening and four attachment holes was bonded to the skull with dental cement (Denta-press, MWdental). The cranial opening was covered with optical paper and attached to the headpiece with peelable adhesive tape (Scotch Magic, 3 M). For retrograde tracing of HVC_X_ neurons, 100 nL (100%) of rhodamine-labeled Retrobeads (Lumfluor Inc., Florida) was injected into Area X. To avoid backflow during injection, pipettes were left in place for 5 min before withdrawal. Craniotomies were covered with Kwik Sil (WPI), and incision sites were sealed with tissue glue. After anesthesia recovery, birds were administered posttreatment meloxicam (0.2 mg/kg body weight) injected into the pectoral muscle for analgesia.

### In Vivo 2-Photon Imaging and Neuron Morphology Analysis.

Imaging of neuronal anatomy was performed using a 2-photon microscope equipped with a galvo–galvo scanner (MOM, Sutter Instruments) coupled to a pulsed Ti:sapphire laser (Mai Tai DeepSee, Spectra-Physics) set to a wavelength of 920 nm and controlled by ScanImage 5.4 software. Brain tissue was exposed to pulsed light with a constant average power of 56 mW. Images were acquired using a 25 × /0.95 N.A. water immersion objective (Leica) and photomultiplier tubes (Hamamatsu). Time-lapse images were taken at different time points relative to DPI 0, the testosterone implantation day. During the recordings, the birds were anesthetized by inhalation of isoflurane and the head was fixed under the 2-photon microscope objective. Images were captured using ScanImage software at 1,024×1,024 pixels and analyzed using ImageJ software. On each DPI, the labeled cells were identified manually. Pairwise distances between cells were calculated using coordinates obtained with ROI Manager in ImageJ. These distances were compared across different DPI imaging sessions. For eGFP-labeled HVCs, in most cases, only one retrogradely labeled HVCx neuron occurred per cell cluster (Fig. 2), excluding an overrepresentation of neighboring neurons in the measurements. For the GCaMP-labeled HVC, we considered only one cell in the distance measurements in case that more than one cell was labeled in a cluster. The measurement error is mainly due to the limited optical resolution along the Z-axis. While the theoretical lateral (XY) resolution is ~480 nm using 920 nm excitation light and 0.95 NA objective, the theoretical axial (Z) resolution is substantially lower, at ~2,000 nm.

For dendritic spines and boutons, 2-photon image stacks were manually aligned, selecting the same segments imaged at different DPI of eGFP-injected females. Images with artifacts were excluded from analysis. Changes in spine and bouton morphology were measured by averaging pixel values across images and subtracting from nonlabeled regions. The numbers of boutons were manually counted. Soma sizes of labeled HVC cells were also analyzed. Given the extended depth of focus for HVC_X_ neuron 2-photon images, the soma sizes were determined by measuring the largest circumference along the z-axis of the cells with ImageJ.

### Modeling of HVC Expansion.

HVC was modeled as a cube with sides of equal length with initial side length (L_0_) of 500 µm. The volume of initial cube is V_0_ = L_0_^3^. Within each cube, 100 points with coordinates [x, y, z] were randomly distributed. The cube volume was expanded by a percentage p ranging from 0% to 100%, resulting in an expanded new volume V′ = V_0_ * (1 + p/100). The new corresponding side length was calculated using uniform linear scaling: L′ = L_0_ * (1 + p/100)^(1/3)^. Thus, the new coordinates [x′, y′, z′] of the 100 points after expansion were computed as [x, y, z] * (1 + p/100)^(1/3)^. Euclidian distances between all pairs of points were calculated for both the initial [x_0_, y_0_, z_0_] and in the expanded [x′, y′, z′] configurations, denoted as [D_0_] and [D′], respectively. Pairwise comparisons of distances between [D_0_] and [D′] were then calculated to quantify the degree of expansion. The initial locations of expansion do not influence the results of the pairwise comparisons. We performed one-way ANOVAs followed by post hoc Tukey tests to compare the expansion models with measured data across all time points and females shown in Fig. 2 and *SI Appendix*, Fig. S4 (*SI Appendix*, Table S2). The q-values were calculated as q = (mean(D_measured_) − mean(D_model_))/SE, where D is the distance value and SE is the standard error of the sum of the means. Positive and negative q-values indicate that measured distances are larger or smaller than model predictions, respectively. Imaging data are available in Edmond (52) and derived data are available in Github (53).

### Histology.

Animals were euthanized with an overdose of isoflurane according to the approved procedure by the government of Upper Bavaria (ROB-55.22532.Vet_02-18-142) between DPI 35 and DPI 42. Brains were removed, snap-frozen on dry ice, and stored at −80 °C for further use. Brains were cryosectioned using a Leica CM3050 cryostat to obtain sections of 20 μm thickness. Five series of parallel sections were mounted on Superfrost5®Plus RNase-free slides with adjacent sections on different slides. Selected sections were mounted onto the 10X Genomics Visium Spatial Gene Expression slides (see below). One series of sections of each bird was Nissl-stained using 0.1% Thionine solution. The HVC sections of the retrogradely traced birds were digitized using a Leica DM6000 B fluorescence microscope before the Nissl staining. We used ImageJ to measure the area size of HVC sections. Slides were numbered with a code so that the analyzing scientist did not know the origin of the digitized sections. HVC volumes of Nissl-stained sections were calculated from the cumulated HVC area size multiplied by the section thickness and the distance between the sections. For comparisons, the area sizes of retrogradely stained and Nissl stained HVC (same section) with the spatial transcriptome-stained HVC (see below), we used sections adjacent to each other, that is sections for spatial transcriptomics were either 20 µm lateral or medial to the Nissl/retrogradely labeled sections. In cases where a section was damaged or lost, the spatial transcriptome section was taken 40 µm away.

### Immunocytochemistry.

To characterize GCaMP-expressing neurons, we performed immunocytochemistry against GFP (part of the GCaMP fusion protein) alongside staining for GABAergic interneurons. Further, HVC_X_ neurons were retrogradely labeled by injection of rhodamine-labeled Retrobeads into Area X (see above). The GCaMP-transfected bird was killed with an overdose of isoflurane and then perfused with phosphate-buffered saline pH 7.4 (PBS), followed by 4% paraformaldehyde in PBS (PFA). The brain was removed and fixed in 4% PFA for 5 to 7 d followed by 10% and 30% PBS-sucrose for 1 d each. Sagittal brain sections (30 µm) were produced with a freezing microtome (CM1325, Leica, Wetzlar, Germany) and every 5th section mounted onto slides and embedded with the antifade mounting medium (VestaShield, Biozol, Eching, Germany). Selected sections were then picked for immunostaining. Sections were washed with 0.5% saponin in PBS, blocked in 10% normal goat serum and 0.5% saponin in PBS, and incubated overnight with the primary antibodies against GFP (1:1,000, chicken, GFP-1020, Aves Labs, Davis, CA) and GAD65/67 (1:200, ABN904, Merck Millipore Corp., MA) in blocking solution at 4 °C. Then, sections were washed with 0.5% saponin in PBS and incubated for 4 h at room temperature with the secondary antibody (goat anti-chicken, conjugated to Alexa Fluor 488, ab150169, Abcam, Cambridge, UK) and (goat anti-rabbit, conjugated to Alexa Fluor 680, A-21109, Invitrogen, MA), respectively, diluted 1:500 in blocking solution. Finally, sections were washed with 0.5% saponin in PBS and embedded with antifade mounting medium on glass slides. The sections were imaged using a confocal microscope (SP5, Leica, Wetzlar, Germany).

### Spatial Transcriptomics.

20-µm sections were loaded onto 10X Genomics Visium Spatial Gene Expression Slides according to the manufacturer’s instructions. The slices were taken between 200 to 250 µm medial from the lateral border of the HVC. The permeabilization conditions were optimized for bird brains using the Visium Spatial Tissue Optimization & Reagent Kit (10X Genomics GmbH, Hamburg). The capture area has 4,992 Spots, 55 µm in diameter each, and a 100 µm center-to-center distance. The libraries of the barcoded cDNA generated from each section were produced following the 10X Genomics Visium library preparation protocol. Indexed libraries were pooled equimolarly and sequenced in a 28/90 paired-end run using the 100 cycles Novaseq sequencing kit v1.5 (#20028316) with NovaSeq XP 2-Lane Kit v1.5 (#20043130) on a Novaseq6000 platform with an S2 flow cell at a minimum of 140 million fragments per sample (140 to 189 M fragments/sample). Demultiplexing was done using the Illumina BCL convert software (v4.2.7.). The raw sequencing reads were processed using SPACE Ranger (10X Genomics (version 2.1.1, 10X Genomics) and mapped to the *S. canaria* reference genome, chromosome-level assembly serCan2020 (NCBI RefSeq accession number GCF_022539315.1). The function aggregate was used to normalize and compare the spatially resolved transcriptomes among birds.

Processing and visualization of the HVC nuclei were performed first using the Loupe browser (7.0.1) to draw and select the HVC barcodes according to the retrograde tracing of HVC (see above). The posterior analysis used the Seurat (5.0.1) (54) and SCTransform (0.4.1) (55) R packages. The 10X Visium spots located within the boundaries of the retrogradely defined HVC boundaries of all sections (one section per animal of four testosterone-treated and four placebo-treated animals) were loaded into Seurat, where the HVC barcode selection was used to subset the sections and create a new Seurat HVC object for each bird, which were merged to perform differential expression analysis across sections. The transcriptomes of one HVC of a testosterone-treated bird and one placebo-treated bird could not be produced due to procedural problems. The SCTransform function was used to normalize the different datasets. Dimensionality reduction was computed through principal-component analysis on the scaled data using the RundPCA function, using the HVC subset of previously determined variable features of the spatial sets as input. As a result, the principal components showed the correlation or anticorrelation of the gene modules across the spots of the datasets. Since the most valid signals are generally captured during the first 10 PCs, they are set as default. The FindNeighbors and FindClusters of the Seurat package were used to calculate a Euclidean distance to the local neighborhoods and to optimize the modularity function (https://satijalab.org/seurat/). The nonlinear reduction UMAP was used to visualize the results. This analysis resulted in just four distinct clusters of transcriptomes within the retrogradely labeled HVCs (*SI Appendix*, Fig. S5).

Finally, the PrepSCTFindMarkers function was used to recorrect the counts of the former transformations, and the DEGs among clusters were assessed using the Seurat FindMarkers function, performing differential expression testing based on the nonparametric Wilcoxon rank sum test. The DEG lists were selected for enrichment analysis (unadjusted *P*-value < 0.05; −0.5 < avg_log2FC > 0.5). *SI Appendix*, Tables S3–S5 list the 100 most significant DEGs; complete lists together with the spatial transcriptome images and selected barcodes are available in Edmond (56). After converting the canary gene symbols to human orthologs, we identified the Gene Ontology (GO) Terms enriched in biological processes and cellular components. We considered all human annotated genes as a reference, using g: Profiler2 R package (57). The graphic visualization of the significant terms (*P*.adjust < 0.00001) was performed using the ggplot function of the ggplot2 R package (58) (R Session info, *SI Appendix*). Due to the small number of spots of cluster 4, those were not further analyzed.

The transcriptome data are available with the BioProject Accession No. PRJNA1212849 (59).

### RNAscope In Situ Hybridization.

HVC sections were fixed in 4% paraformaldehyde for 1 h at 4 °C. In situ hybridizations were performed using the RNAscope® Multiplex Fluorescent Detection Reagents—v2 Kit (Cat. No. 323110, Advanced Cell Diagnostics, Newark, CA), in accordance with the manufacturer’s protocol. AR mRNA was detected with an AR-probe (Accession No. NM_001076688.1; Tgu_AR_C1/C2, Cat. No.469741-C1/C2), and ESR1 mRNA, was detected with an ESR1 probe (Accession No. NM_001076701.1; Tgu_ESR1, Cat. No. 488021-C1). Additionally, a CCK probe was designed to detect CCK mRNA (Accession No. XM_009086203.4; Sc-CCK-C3, Cat. No. 1824191-C3). For quality control, positive and negative control probes, PPIB (Cat. No. 460351) and DAPB (Cat. No. 310043), respectively, were included in the assay (Advanced Cell Diagnostics).

### Song Recording and Analysis.

Each testosterone-treated female was housed with an untreated female canary within custom-made soundproof chambers (120 × 50 × 50 cm). Each chamber was equipped with a microphone (TC20, Earthworks). The sounds were recorded digitally with a sampling rate of 44.1 kHz and an accuracy of 16 bits using SAP2011 software. Custom MATLAB scripts were developed to isolate bird songs from the recorded audio tracks and sound features including duration, amplitude, pitch, frequency modulation, amplitude modulation, mean frequency, harmonic ratio, spectral-Centroid, -Spread, -Skewness, -Kurtosis, -Flux, -Rolloff point, -Slope, -Flatness, -Decrease, and -Crest of each syllable were extracted from the bird songs using MATLAB’s audio toolbox, specifically its spectral descriptor functions in the R2021b version. We analyzed 186 subsongs including 3,140 syllables, 871 plastic-songs including 26,510 syllables, and 907 full songs including 45,151 syllables of the 10 testosterone-treated females. We monitored vocal behavior during the entire song development to assure that each bird reached the stage of full songs and to determine at which DPI. Details of the song development will be published elsewhere.

## Supplementary Material

Appendix 01 (PDF)

## Data Availability

The transcriptome data are available with the BioProject Accession No. PRJNA1212849 (59). Spatial transcriptome images and selected barcodes are available in Edmond — the Open Research Data Repository of the Max Planck Society (56). The 2-photon images of HVCs are available in Edmond (52), and derived data are available in GitHub (53). All other data are included in the manuscript and/or *SI Appendix*.
