# Supplementary material for: Invariant HVC size in female canaries singing under testosterone: Unlocking function through neural differentiation, not growth
Source: Proc Natl Acad Sci U S A. 2025 Oct 20;122(43):e2426847122. doi: 10.1073/pnas.2426847122 (PMC12582222; doi:10.1073/pnas.2426847122)
Supplement: Supplementary file 1 — Appendix 01 (PDF) [file pnas.2426847122.sapp.pdf]

Supplementary Information:

Invariant HVC size in female canaries singing under testosterone: Unlocking function through neural differentiation, not growth.

Shouwen Ma, Carolina Frankl-Vilches, Manfred Gahr*

Supplementary Tables and Figures:

5 Tables

10 Figures

Supplementary Tab. S1: HVC area size defined by cytoarchitecture (Nissl-stain), connectivity (retrograde tracing) and molecular markers (spatial transcriptomics, ST). Area sizes are in µm^2. ST area is that of Cluster 1 in testosterone-treated and of Cluster 2 in placebo-treated females. ST could not be analyzed in one testosterone-treated female (section partially destroyed) and in one placebo-treated female (technical problem with transcriptome). We further calculated the rations in [%] of the sizes of traced, Nissl-stained and ST derived HVC areas (see Fig. 3). Trace/Nissl rations were significantly different between testosterone-treated and placebo-treated animals ($t(6) = -6.13$, $p = 0.0004$) while ST/Nissl ratios of these groups are not different ($t(4) = 0.81$, $p = 0.24$). ST/Nissl ratios and ST/Trace ratios of testosterone-treated females were similar (($t(4) = 1.55$, $p = 0.10$) while that of placebo-treated females were significantly different (($t(4) = 7.23$, $p = 0.001$). Since the cutting angles between the brains are not exactly the same, this could lead to small differences in the size of the HVC areas. Therefore, we calculated the ratios of the measured areas for comparisons between the groups. n.d. = no data.

Hormone treatment	Nissl (µm2)	Tracing (µm2)	ST (µm2)	Trace/Nissl (%)	ST/Nissl (%)	ST/Trace (%)
Testo	117405	143099	108955	121.9	92.8	76.1
Testo	138424	150378	125554	108.6	90.7	83.5
Testo	159597	178431	n.d.	111.8		
Testo	139405	135743	124450	97.4	89.3	91.7
Mean	138708	151913	119653	109.9	90.9	83.8
SD	17231	18661	9281	10.1	1.8	7.8
Placebo	90026	134824	80314	149.8	89.2	59.6
Placebo	106017	154321	n.d.	145.6		
Placebo	72466	119320	67517	164.7	93.2	56.6
Placebo	79013	134753	65107	170.5	82.4	48.3
Mean	86881	135805	70979	157.6	88.3	54.8
SD	14672	14337	8173	11.9	5.4	5.8

Supplementary Tab. S2. The observed changes in pairwise cell distances derived from in-vivo 2-photon imaging of HVC were compared to data sets corresponding to 0%, 2%, 3%, 5% and 10% expansion models of HVC. Note that most observed data were different from 5% expansion and all observed data were different from 10% expansion models. We performed one-way ANOVAs for each expansion model [$f(1,93) = 73.5$, $p < 0.001$] for all observation days of the five females (F8, F29, F32, F41, F112) of Fig. 2; and for female F18 of Supplementary Fig. S3 [$f(1,25) = 86.6$, $p < 0.001$]. The listed p-values relate to the Tukey post hoc tests for all observation days of all animals and the 0%, 2%, 3%, 5% and 10% expansion models. The q-values show the direction of the changes, with negative q-values indicating a decrease and positive q-values indicating an increase in pairwise cell distances. Bird ID relates to the studied animals. DPI is the day after testosterone implantation at which the pairwise distances were observed; mean and sd (standard deviation) are observed changes of pairwise distances at the various DPIs of the females. P-values that were similar to the models of 2%, 3%, 5% and 10% expansion were indicated as yellow (negative q value) and orange (positive q value). The data are available at https://github.com/ShouwenMa/femaleCanaries/tree/main/ Analysis/matlabCode.

Bird ID	DPI	Change in pairwise distance		Post-hoc Tukey tests comparing HVC expansion models and observed data									
				0%	0%	2%	2%	3%	3%	5%	5%	10%	10%
		mean (µm)	sd (µm)	q	p	q	p	q	p	q	p	q	p
F8	0	-0.090	4.909	1.08	1.00	-9.33	0.01	-13.76	0.00	-22.53	0.00	-43.99	0.00
F8	7	0.661	2.794	36.22	0.02	-51.59	0.00	-88.94	0.00	-162.92	0.00	-343.85	0.00
F8	9	-0.777	1.908	-18.80	0.55	-124.19	0.00	-169.01	0.00	-257.80	0.00	-474.93	0.00
F8	12	-1.550	5.135	-23.39	0.25	-70.55	0.00	-90.61	0.00	-130.34	0.00	-227.51	0.00
F8	15	1.807	5.804	30.63	0.01	-4.03	1.00	-18.77	0.28	-47.97	0.00	-119.38	0.00
F8	23	0.092	2.103	11.68	0.74	-53.64	0.00	-81.42	0.00	-136.45	0.00	-271.02	0.00
F8	26	1.374	2.138	41.60	0.00	-17.33	0.20	-42.39	0.00	-92.04	0.00	-213.45	0.00
F8	30	-0.028	2.035	6.92	0.95	-46.51	0.00	-69.24	0.00	-114.25	0.00	-224.33	0.00
F29	0	-4.044	3.207	-33.42	0.00	-57.19	0.00	-66.84	0.00	-85.95	0.00	-132.69	0.00
F29	3	0.951	4.762	8.89	0.18	-7.19	0.43	-13.71	0.00	-26.64	0.00	-58.26	0.00
F29	6	3.429	5.414	18.84	0.00	6.49	0.36	1.47	1.00	-8.46	0.09	-32.75	0.00
F29	11	2.695	3.564	20.75	0.00	3.98	0.91	-2.83	0.98	-16.31	0.00	-49.28	0.00
F29	14	0.622	4.735	5.08	0.58	-6.90	0.21	-11.77	0.00	-21.41	0.00	-44.98	0.00
F29	18	0.171	3.260	1.08	0.99	-3.28	0.29	-5.04	0.01	-8.54	0.00	-17.09	0.00
F29	22	1.482	3.753	4.24	0.16	-1.33	0.99	-3.59	0.34	-8.07	0.00	-19.02	0.00
F29	27	-1.467	3.625	-1.71	0.91	-6.04	0.00	-7.80	0.00	-11.28	0.00	-19.79	0.00
F29	32	2.574	1.505	4.50	0.02	0.72	1.00	-0.82	1.00	-3.86	0.08	-11.29	0.00
F29	35	0.806	1.461	0.59	1.00	-0.59	1.00	-1.07	0.95	-2.02	0.46	-4.34	0.00

Bird	DPI	Change in pairwise		Post-hoc Tukey tests comparing HVC expansion models and observed data									
ID		distance		0%	0%	2%	2%	3%	3%	5%	5%	10%	10%
		mean (µm)	sd (µm)	q	p	q	p	q	p	q	p	q	p
F32	0	-2.406	4.653	-8.02	0.02	-17.48	0.00	-21.50	0.00	-29.47	0.00	-48.96	0.00
F32	2	3.347	2.595	11.23	0.00	3.83	0.47	0.68	1.00	-5.56	0.08	-20.83	0.00
F32	5	0.030	1.940	1.15	1.00	-6.37	0.02	-9.57	0.00	-15.91	0.00	-31.41	0.00
F32	8	1.466	3.009	4.12	0.19	-1.42	0.98	-3.78	0.28	-8.46	0.00	-19.89	0.00
F32	13	0.096	2.108	0.89	1.00	-4.06	0.14	-6.16	0.00	-10.34	0.00	-20.54	0.00
F32	16	0.348	1.887	1.30	0.98	-3.27	0.33	-5.22	0.01	-9.07	0.00	-18.49	0.00
F32	20	-0.096	2.032	0.43	1.00	-3.85	0.13	-5.67	0.00	-9.27	0.00	-18.09	0.00
F32	23	-0.558	2.034	-0.27	1.00	-3.35	0.13	-4.66	0.01	-7.25	0.00	-13.60	0.00
F32	28	0.428	1.626	0.62	1.00	-1.34	0.90	-2.17	0.47	-3.81	0.01	-7.84	0.00
F32	33	1.494	4.722	1.86	0.72	-0.61	1.00	-1.66	0.82	-3.74	0.03	-8.82	0.00
F41	0	1.863	4.971	14.29	0.00	-1.36	1.00	-7.56	0.34	-19.84	0.00	-49.86	0.00
F41	8	6.073	3.889	133.74	0.00	80.75	0.00	59.77	0.00	18.22	0.34	-83.40	0.00
F41	11	5.510	3.384	137.44	0.00	77.90	0.00	54.33	0.00	7.64	0.98	-106.53	0.00
F41	13	-0.659	2.814	-2.45	1.00	-50.07	0.00	-68.92	0.00	-106.25	0.00	-197.56	0.00
F41	18	-1.612	2.235	-42.12	0.00	-143.30	0.00	-183.35	0.00	-262.69	0.00	-456.71	0.00
F41	21	-5.460	3.484	-125.85	0.00	-192.49	0.00	-218.88	0.00	-271.13	0.00	-398.93	0.00
F41	27	-6.096	3.920	-96.42	0.00	-141.65	0.00	-159.56	0.00	-195.02	0.00	-281.76	0.00
F41	34	-4.602	4.046	-28.77	0.00	-47.21	0.00	-54.51	0.00	-68.97	0.00	-104.34	0.00
F41	40	-3.163	4.022	-13.78	0.00	-27.42	0.00	-32.82	0.00	-43.52	0.00	-69.69	0.00
F112	0	-1.033	2.304	-10.84	0.39	-46.39	0.00	-61.76	0.00	-92.20	0.00	-166.66	0.00
F112	2	0.268	1.833	13.53	0.43	-43.16	0.00	-67.67	0.00	-116.22	0.00	-234.96	0.00
F112	4	0.119	1.835	8.90	0.80	-41.55	0.00	-63.36	0.00	-106.57	0.00	-212.24	0.00
F112	8	3.230	3.308	59.55	0.00	19.22	0.06	1.78	1.00	-32.76	0.00	-117.25	0.00
F112	12	4.026	2.460	79.87	0.00	35.72	0.00	16.62	0.11	-21.19	0.01	-113.68	0.00
F112	15	2.320	1.987	47.48	0.00	4.16	0.99	-14.57	0.16	-51.68	0.00	-142.43	0.00
F112	18	5.308	3.395	74.56	0.00	42.76	0.00	29.01	0.00	1.77	1.00	-64.84	0.00
F112	22	0.413	2.670	10.75	0.46	-25.16	0.00	-40.69	0.00	-71.45	0.00	-146.67	0.00
F112	26	2.091	4.013	21.74	0.00	0.01	1.00	-9.38	0.35	-27.99	0.00	-73.49	0.00
F112	33	0.072	5.005	1.33	1.00	-7.17	0.04	-10.85	0.00	-18.14	0.00	-35.95	0.00
F18	5	-2.853	5.688	-12.78	0.00	-26.63	0.00	-32.24	0.00	-43.36	0.00	-70.55	0.00
F18	7	1.075	4.651	9.12	0.11	-6.06	0.57	-12.21	0.01	-24.40	0.00	-54.19	0.00
F18	11	-1.142	4.661	-3.92	0.91	-19.19	0.00	-25.37	0.00	-37.62	0.00	-67.58	0.00
F18	13	-0.288	2.639	1.37	1.00	-16.92	0.00	-24.33	0.00	-39.00	0.00	-74.90	0.00
F18	17	0.927	3.552	9.01	0.12	-7.83	0.25	-14.66	0.00	-28.18	0.00	-61.25	0.00
F18	20	2.381	5.598	15.60	0.00	1.71	1.00	-3.92	0.91	-15.07	0.00	-42.35	0.00
F18	21	2.339	8.175	11.75	0.01	1.08	1.00	-3.25	0.96	-11.82	0.01	-32.77	0.00
F18	24	-1.479	8.487	-3.96	0.91	-14.52	0.00	-18.79	0.00	-27.27	0.00	-47.99	0.00
F18	26	-0.408	3.808	0.18	1.00	-16.31	0.00	-23.00	0.00	-36.24	0.00	-68.63	0.00
F18	30	-0.205	3.218	1.80	1.00	-15.45	0.00	-22.44	0.00	-36.28	0.00	-70.14	0.00
F18	32	0.699	3.564	7.23	0.34	-9.57	0.08	-16.38	0.00	-29.86	0.00	-62.84	0.00
F18	35	-1.461	3.581	-5.82	0.62	-22.71	0.00	-29.55	0.00	-43.11	0.00	-76.27	0.00
F18	38	-0.323	2.047	1.01	1.00	-18.04	0.00	-25.76	0.00	-41.05	0.00	-78.44	0.00

Supplementary Tab. S3. The differential gene expression (DEG) between Cluster 1 and 3, accessed through the Seurat's Find Markers function (see methods). The resulting data frame shows the following columns: p_val: p-value (unadjusted), avg_log2FC (log fold-change of the average expression between the two clusters); pct.1 (the percentage of spots where the feature is detected in Cluster 1, pct.2 (the percentage of spots where the feature is detected in the Cluster 3), and p_val_adj (adjusted p-value, based on Bonferroni correction using all features in the dataset). The 100 genes with the most significant p-val are shown, sorted according to the highest avg_log2F differences. A positive avg_log2F means higher expression in Cluster 1. The values for the ESR1 gene are also listed.

gene	p_val		avg_log2FC	pct.1	pct.2		p_val_adj
CADPS2	2.09E-11		3.358	0.767	0.125		2.89E-07
SYT2	5.78E-10		2.905	0.767	0.179		7.98E-06
LOC103817657	7.06E-12		2.867	0.86	0.179		9.75E-08
EMILIN2	6.08E-09		2.459	0.674	0.089		8.39E-05
ALDH1A2	1.60E-10		2.293	0.884	0.268		2.20E-06
SRD5A2	4.67E-10		2.293	0.86	0.304		6.44E-06
LOC103819056	2.78E-07		2.222	0.674	0.196		3.84E-03
AR	4.32E-06		2.107	0.628	0.196		5.96E-02
LOC103819003	7.01E-06		2.033	0.488	0.071		9.68E-02
TENM1	4.09E-09		2.004	0.86	0.357		5.65E-05
CAMK1D	1.91E-11		1.931	1	0.696		2.63E-07
UTS2B	4.27E-09		1.879	0.93	0.464		5.90E-05
IGFBP5	4.07E-06		1.825	0.721	0.321		5.62E-02
FGF1	5.35E-07		1.629	0.837	0.429		7.38E-03
SESN3	9.51E-07		1.614	0.837	0.482		1.31E-02
PVALB	7.38E-15		1.612	1	0.857		1.02E-10
BMERB1	2.47E-06		1.591	0.767	0.446		3.41E-02
EDIL3	5.11E-10		1.579	0.977	0.768		7.06E-06
LMO3	3.81E-08		1.572	0.907	0.589		5.25E-04
NT5DC1	4.39E-06		1.539	0.767	0.339		6.06E-02
TRPM2	4.72E-09		1.514	1	0.625		6.52E-05
C2CD2L	4.91E-06		1.490	0.791	0.411		6.78E-02
SH3GLB2	9.97E-09		1.420	1	0.589		1.38E-04
PCDH7	2.29E-07		1.381	0.93	0.607		3.15E-03
SATB1	7.78E-06		1.381	0.814	0.464		1.07E-01
CNTNAP1	3.77E-06		1.353	0.814	0.339		5.20E-02
FOSL2	6.75E-08		1.331	0.93	0.589		9.31E-04
BAG5	5.55E-07		1.308	0.977	0.625		7.66E-03
LOC108962171	4.41E-06		1.274	0.837	0.411		6.09E-02
FAM20C	3.52E-10		1.266	1	0.857		4.86E-06
SEMA5A	3.37E-06		1.232	0.907	0.571		4.66E-02
DSEL	5.27E-06		1.229	0.837	0.429		7.28E-02
ADAM23	3.05E-06		1.176	0.93	0.661		4.21E-02
LURAP1	6.37E-06		1.109	0.977	0.625		8.79E-02

NTS	3.62E-10		1.086	1	0.964		4.99E-06
DTNBP1	4.91E-06		1.085	0.953	0.607		6.77E-02
NEFL	2.75E-12		1.078	1	0.982		3.80E-08
YPEL5	5.39E-11		1.073	1	0.911		7.44E-07
NEFM	4.01E-07		1.024	1	0.804		5.53E-03
SCRN1	6.85E-07		1.020	0.977	0.768		9.46E-03
LAPTM4B	2.48E-06		1.013	0.953	0.75		3.42E-02
LOC103823432	2.00E-06		0.970	0.977	0.857		2.76E-02
NSF	1.56E-10		0.954	1	0.946		2.15E-06
RAB11FIP2	5.03E-06		0.915	0.977	0.839		6.94E-02
CLTA	3.89E-06		0.875	0.977	0.875		5.36E-02
PPP3R1	6.43E-11		0.840	1	1		8.88E-07
FAM131B	1.01E-05		0.819	1	0.839		1.39E-01
FKBP4	3.91E-07		0.796	1	0.946		5.40E-03
LOC103817575	6.94E-06		0.756	0.977	0.964		9.57E-02
TUBA1B	1.78E-12		0.752	1	1		2.45E-08
RGS4	3.50E-09		0.715	1	0.982		4.83E-05
LOC103826028	2.83E-07		0.699	1	1		3.90E-03
NAPB	2.67E-10		0.696	1	1		3.69E-06
PKM	5.37E-09		0.666	1	1		7.41E-05
STMN3	3.56E-09		0.645	1	1		4.92E-05
OAZ1	5.49E-11		0.614	1	1		7.57E-07
GPI	3.02E-06		0.609	1	0.964		4.17E-02
SERPINI1	3.01E-08		0.608	1	1		4.15E-04
CHGB	1.83E-06		0.589	1	1		2.53E-02
EIF5	5.14E-06		0.573	1	0.982		7.10E-02
SOD1	6.27E-08		0.549	1	1		8.65E-04
YWHAG	8.84E-07		0.533	1	1		1.22E-02
MDH1	3.26E-06		0.518	1	1		4.50E-02
SNCB	3.24E-07		0.498	1	1		4.47E-03
NRGN	4.57E-07		0.489	1	1		6.30E-03
TSPAN7	1.78E-07		0.489	1	1		2.45E-03
EIF1	1.20E-07		0.432	1	1		1.66E-03
RTN1	2.13E-07		0.394	1	1		2.95E-03
LOC103816871	5.57E-06		0.385	1	1		7.69E-02
LOC108963315	4.07E-06		0.365	1	1		5.62E-02
ATP5MC3	3.17E-06		0.338	1	1		4.37E-02
RPS24	4.69E-06		-0.416	1	1		6.48E-02
RPS29	1.53E-08		-0.482	1	1		2.11E-04
RPS20	4.62E-06		-0.515	1	1		6.38E-02
UBA52	9.63E-08		-0.515	1	1		1.33E-03
DDH36-mgp13	7.82E-06		-0.524	1	1		1.08E-01
RPL27	3.63E-09		-0.530	1	1		5.00E-05
RPL34	7.56E-07		-0.542	1	1		1.04E-02
RPL14	1.79E-06		-0.546	1	1		2.47E-02
RPL9	1.06E-06		-0.575	1	1		1.47E-02
DDH36-mgp08	8.12E-07		-0.583	1	1		1.12E-02
RPS7	1.03E-05		-0.602	1	1		1.42E-01

RPL32	5.36E-06		-0.604	1	1		7.40E-02
RPS23	9.55E-11		-0.605	1	1		1.32E-06
RPS25	1.37E-06		-0.647	1	1		1.88E-02
DDH36-mgr02	1.78E-09		-0.692	1	1		2.46E-05
LOC127059190	1.17E-06		-0.852	0.977	1		1.62E-02
PCP4	6.86E-06		-0.910	0.884	1		9.47E-02
MARCKS	8.95E-06		-1.154	0.674	0.929		1.24E-01
SNCA	7.54E-10		-1.170	1	1		1.04E-05
SH3BGRL	1.27E-06		-1.188	0.767	0.946		1.75E-02
RPLP1	3.49E-07		-1.367	0.907	0.929		4.82E-03
ARHGDIB	1.69E-06		-1.457	0.581	0.875		2.34E-02
LOC127060513	3.26E-09		-1.495	0.628	0.982		4.51E-05
IGFBP7	7.92E-06		-1.609	0.558	0.821		1.09E-01
OST4	7.79E-07		-2.008	0.581	0.839		1.08E-02
SCG2	2.88E-08		-2.300	0.256	0.804		3.97E-04
VIM	4.01E-06		-2.339	0.512	0.821		5.54E-02
LOC103817276	9.08E-09		-2.549	0.395	0.804		1.25E-04
CCK	2.37E-10		-3.173	0.256	0.857		3.27E-06
ESR1	0.001715		1.488	0.442	0.161		1.00E+00

Supplementary Tab. S4. The differential gene expression (DEG) between Cluster 2 and 3, accessed through the Seurat's Find Markers function (see methods). The resulting data frame shows the following columns: p_val: p-value (unadjusted), avg_log2FC (log fold-change of the average expression between the two clusters); pct.1 (the percentage of spots where the feature is detected in Cluster 2, pct.2 (the percentage of spots where the feature is detected in the Cluster 3), and p_val_adj (adjusted p-value, based on Bonferroni correction using all features in the dataset). The 100 genes with the most significant p-val are shown, sorted according to the highest avg_log2F differences. A positive avg_log2F means higher expression in Cluster 2. The values for the AR and ESR1 gene are also listed.

gene	p_val		avg_log2FC	pct.1	pct.2		p_val_adj
SLN	2.36E-06		4.907	0.357	0		3.26E-02
GFRA1	3.45E-09		4.459	0.571	0.018		4.76E-05
IGF2	2.62E-07		4.248	0.464	0.018		3.62E-03
PHF2	3.67E-04		3.807	0.214	0		1.00E+00
TMEM216	5.75E-05		3.459	0.321	0.018		7.93E-01
LOC103817657	3.83E-09		3.451	0.786	0.179		5.28E-05
CADPS2	8.17E-10		3.000	0.786	0.125		1.13E-05
UTS2B	1.16E-11		2.888	1	0.464		1.61E-07
POLQ	7.99E-05		2.874	0.321	0.018		1.00E+00
ST6GALNAC1	2.70E-04		2.874	0.321	0.036		1.00E+00
TMEM130	2.77E-04		2.807	0.357	0.054		1.00E+00
SV2B	2.84E-04		2.737	0.321	0.036		1.00E+00
TMEM233	2.77E-04		2.700	0.357	0.054		1.00E+00
LOC103819056	1.70E-08		2.585	0.786	0.196		2.35E-04
SLC25A4	3.22E-04		2.459	0.357	0.054		1.00E+00
NAA60	3.22E-04		2.459	0.357	0.054		1.00E+00
PALS1	2.28E-05		2.415	0.5	0.089		3.15E-01
SRD5A2	4.88E-07		2.131	0.786	0.304		6.73E-03
SMG8	3.27E-04		2.100	0.429	0.089		1.00E+00
TMEM200B	2.53E-04		2.000	0.464	0.107		1.00E+00
TENM1	2.43E-06		1.941	0.786	0.357		3.36E-02
LOC103818208	3.15E-04		1.907	0.464	0.107		1.00E+00
SYT2	6.92E-05		1.874	0.607	0.179		9.55E-01
EMILIN2	1.64E-04		1.830	0.464	0.089		1.00E+00
FAM149A	1.80E-04		1.807	0.571	0.179		1.00E+00
MED24	3.12E-04		1.766	0.536	0.161		1.00E+00
ID2	5.92E-07		1.626	0.893	0.589		8.18E-03
PSMD6	2.34E-04		1.524	0.643	0.232		1.00E+00
GTF2H5	8.23E-09		1.472	1	0.875		1.14E-04
NT5DC1	3.33E-06		1.469	0.857	0.339		4.60E-02
DHCR7	1.61E-04		1.459	0.679	0.25		1.00E+00
NEFM	2.74E-08		1.453	1	0.804		3.78E-04
MCTP1	2.44E-05		1.402	0.857	0.411		3.37E-01

BCAS2	3.58E-08		1.394	0.964	0.839		4.94E-04
CNTNAP1	1.04E-04		1.387	0.786	0.339		1.00E+00
SESN3	2.33E-04		1.379	0.786	0.482		1.00E+00
LOC108962171	2.25E-05		1.363	0.857	0.411		3.10E-01
LOC103822176	3.39E-05		1.356	0.821	0.339		4.68E-01
DPP6	2.42E-08		1.335	0.964	0.839		3.34E-04
FGF1	3.49E-04		1.285	0.786	0.429		1.00E+00
PVALB	1.05E-05		1.279	1	0.857		1.45E-01
ENO2	8.12E-07		1.252	1	0.75		1.12E-02
LOC103824724	3.37E-04		1.227	0.821	0.464		1.00E+00
TIMP2	7.62E-06		1.193	1	0.786		1.05E-01
LOC103822992	1.80E-04		1.175	0.821	0.411		1.00E+00
TRPM2	5.75E-05		1.158	0.929	0.625		7.94E-01
FLRT2	4.02E-05		1.093	0.929	0.589		5.54E-01
CAMK1D	3.25E-04		1.080	0.929	0.696		1.00E+00
SCRN1	5.84E-05		1.048	0.964	0.768		8.07E-01
NEFL	1.78E-07		0.987	1	0.982		2.46E-03
YPEL5	1.27E-06		0.979	1	0.911		1.75E-02
LOC103823960	6.36E-06		0.972	1	0.911		8.78E-02
LOC103823432	8.46E-05		0.925	1	0.857		1.00E+00
NELL2	2.31E-06		0.914	1	0.982		3.19E-02
ZEB2	1.11E-04		0.906	1	0.875		1.00E+00
NDUFA5	1.73E-09		0.840	1	1		2.39E-05
RGS4	3.86E-07		0.793	1	0.982		5.33E-03
C4H4orf48	9.67E-05		0.737	1	0.911		1.00E+00
NTS	5.07E-05		0.714	1	0.964		7.00E-01
OAZ1	7.48E-10		0.681	1	1		1.03E-05
DDH36-mgp06	9.50E-07		0.604	1	1		1.31E-02
LOC103823737	1.04E-04		0.594	1	1		1.00E+00
SERPINI1	2.98E-06		0.558	1	1		4.12E-02
OLFM1	1.82E-04		0.459	1	1		1.00E+00
GPX4	2.56E-04		0.438	1	1		1.00E+00
DDH36-mgp13	4.04E-05		0.435	1	1		5.57E-01
DDH36-mgp10	4.97E-05		0.423	1	1		6.87E-01
HINT1	1.41E-04		0.417	1	1		1.00E+00
LOC103821991	2.09E-05		0.409	1	1		2.88E-01
DDH36-mgp07	3.99E-06		0.409	1	1		5.51E-02
TUBA1B	2.72E-04		0.404	1	1		1.00E+00
ATP5F1E	8.57E-05		0.353	1	1		1.00E+00
DBI	3.54E-04		0.261	1	1		1.00E+00
RPL37A	6.93E-05		-0.335	1	1		9.57E-01
RPL23	1.61E-04		-0.448	1	1		1.00E+00
GPM6A	9.56E-05		-0.457	1	1		1.00E+00
RPL7	1.15E-04		-0.465	1	1		1.00E+00
ACTB	9.08E-06		-0.472	1	1		1.25E-01
GPM6B	2.46E-04		-0.496	1	1		1.00E+00

STMN1	1.00E-06		-0.519	1	1		1.38E-02
ACTG1	1.11E-05		-0.570	1	1		1.54E-01
CIRBP	2.02E-04		-0.742	1	1		1.00E+00
CAMK2N1	9.48E-08		-0.808	1	1		1.31E-03
EEF1D	2.63E-04		-0.883	0.929	0.964		1.00E+00
SYNPR	3.48E-06		-0.955	0.929	1		4.81E-02
NSG2	1.10E-06		-0.967	1	1		1.52E-02
AGT	7.58E-05		-0.983	1	0.964		1.00E+00
MEF2A	2.17E-04		-1.070	0.679	0.893		1.00E+00
PLCB1	8.65E-07		-1.100	0.964	1		1.19E-02
CLU	3.31E-05		-1.141	0.786	1		4.56E-01
APOA1	6.39E-06		-1.200	0.5	0.929		8.82E-02
LOC103826946	1.99E-04		-1.237	0.857	0.946		1.00E+00
HSP90B1	1.95E-06		-1.269	0.679	0.964		2.69E-02
KCNG1	8.24E-05		-1.467	0.357	0.804		1.00E+00
ARHGDIB	7.27E-06		-1.636	0.464	0.875		1.00E-01
MARCKS	1.75E-07		-1.828	0.536	0.929		2.42E-03
CCK	3.79E-05		-2.019	0.607	0.857		5.24E-01
LSP1	2.40E-04		-2.209	0.107	0.518		1.00E+00
NR4A1	7.26E-06		-4.322	0	0.5		1.00E-01
FOS	9.41E-05		-4.358	0	0.411		1.00E+00
AR	2.32E-01		0.621	0.321	0.196		1.00E+00
ESR1	2.46E-01		-1.115	0.071	0.161		1.00E+00

Supplementary Tab. S5. The differential gene expression (DEG) between Cluster 1 and 2, accessed through the Seurat's Find Markers function (see methods). The resulting data frame shows the following columns: p_val: p-value (unadjusted), avg_log2FC (log fold-change of the average expression between the two clusters); pct.1 (the percentage of spots where the feature is detected in Cluster 1, pct.2 (the percentage of spots where the feature is detected in the Cluster 2), and p_val_adj (adjusted p-value, based on Bonferroni correction using all features in the dataset). The 100 genes with the most significant p-val are shown, sorted according to the highest avg_log2F differences. A positive avg_log2F means. higher expression in Cluster 1. The values for the AR, CCK and ESR1 gene are also listed

gene	p_val		avg_log2FC	pct.1	pct.2		p_val_adj
LOC103825100	1.80E-05		3.136	0.535	0.036		0.248
RIDA	8.79E-07		3.006	0.674	0.071		0.012
S100B	7.91E-06		2.640	0.698	0.214		0.109
ZFHX4	1.83E-05		2.066	0.721	0.214		0.252
IGFBP5	5.39E-05		2.009	0.721	0.25		0.744
PMM1	1.50E-05		1.729	0.814	0.321		0.207
EDIL3	8.63E-08		1.608	0.977	0.857		0.001
ABI2	1.50E-05		1.559	0.86	0.536		0.207
RIC8A	1.18E-04		1.536	0.767	0.321		1.000
HSP90B1	3.70E-07		1.427	0.977	0.679		0.005
PCMT1	2.39E-06		1.381	0.953	0.607		0.033
PHF24	3.27E-07		1.356	1	0.857		0.005
CTSB	4.02E-07		1.334	1	0.75		0.006
TF	1.10E-06		1.322	0.977	0.893		0.015
LOC103814303	1.69E-05		1.296	0.93	0.714		0.234
LOC103826946	4.86E-06		1.283	0.953	0.857		0.067
DDX3X	1.18E-04		1.260	0.907	0.571		1.000
CLU	8.40E-07		1.232	0.953	0.786		0.012
APOA1	6.84E-05		1.198	0.907	0.5		0.945
ATP2B4	1.71E-07		1.173	1	0.929		0.002
DHCR24	7.92E-05		1.147	0.93	0.571		1.000
LAPTM4B	1.08E-04		1.087	0.953	0.679		1.000
PFKL	4.73E-05		1.070	0.953	0.679		0.653
TUSC3	1.23E-04		1.063	0.884	0.571		1.000
LOC103816329	3.05E-06		0.979	1	1		0.042
CIRBP	9.52E-08		0.953	1	1		0.001
AGT	9.60E-07		0.948	1	1		0.013
UBC	4.11E-06		0.887	1	0.964		0.057
GNG2	1.15E-04		0.835	1	0.964		1.000
PTN	5.56E-05		0.801	1	0.964		0.768
SNCB	2.26E-09		0.703	1	1		0.000
GPM6A	9.12E-08		0.703	1	1		0.001

YWHAG	2.75E-07		0.674	1	1		0.004
LOC103826028	3.81E-05		0.670	1	1		0.526
PINK1	1.86E-05		0.646	1	1		0.256
CAMK2N1	6.62E-08		0.621	1	1		0.001
PEBP1	3.46E-05		0.617	1	1		0.477
VDAC3	9.18E-05		0.609	1	1		1.000
LOC103819224	2.34E-06		0.601	1	1		0.032
SERINC1	5.38E-07		0.594	1	1		0.007
ATF4	3.80E-05		0.582	1	1		0.524
ACTB	4.38E-07		0.554	1	1		0.006
GALNT17	1.29E-04		0.523	1	1		1.000
PPP3R1	5.36E-05		0.502	1	1		0.739
ACTG1	1.19E-04		0.500	1	1		1.000
NAPB	2.52E-05		0.470	1	1		0.348
CLSTN1	4.07E-05		0.456	1	1		0.561
PRDX1	3.16E-05		0.452	1	1		0.436
TUBB4B	3.80E-05		0.451	1	1		0.524
STMN1	1.06E-05		0.444	1	1		0.146
PKM	8.96E-05		0.434	1	1		1.000
LOC103816871	4.94E-06		0.424	1	1		0.068
NRGN	1.43E-04		0.408	1	1		1.000
YWHAZ	6.66E-05		0.394	1	1		0.920
SOD1	9.51E-05		0.379	1	1		1.000
TUBA1B	6.10E-05		0.348	1	1		0.841
HSP90AA1	2.79E-06		0.343	1	1		0.039
HSPA8	1.00E-04		0.237	1	1		1.000
RPS24	6.22E-05		-0.345	1	1		0.858
DDH36-mgr01	7.10E-06		-0.368	1	1		0.098
RPS29	5.47E-07		-0.451	1	1		0.008
RPL32	1.04E-04		-0.455	1	1		1.000
DDH36-mgp11	7.86E-09		-0.486	1	1		0.000
MLLT11	2.49E-05		-0.539	1	1		0.343
UBA52	2.43E-07		-0.545	1	1		0.003
NDUFA5	3.92E-06		-0.553	1	1		0.054
ACBD7	4.70E-05		-0.569	1	1		0.649
RPL9	8.32E-06		-0.592	1	1		0.115
RPS7	4.59E-07		-0.600	1	1		0.006
DDH36-mgp07	1.38E-10		-0.648	1	1		0.000
RPS28	1.14E-05		-0.670	0.977	1		0.158
DDH36-mgr02	5.34E-10		-0.678	1	1		0.000
DDH36-mgp03	3.10E-10		-0.751	1	1		0.000
RPS25	1.59E-08		-0.773	1	1		0.000
DDH36-mgp02	9.31E-10		-0.796	1	1		0.000
DDH36-mgp04	6.79E-09		-0.865	1	1		0.000
DDH36-mgp08	9.24E-11		-0.873	1	1		0.000

DDH36-mgp10	1.21E-11		-0.930	1	1		0.000
DDH36-mgp06	5.99E-12		-0.941	1	1		0.000
DDH36-mgp13	1.97E-11		-0.960	1	1		0.000
FRMD8	4.05E-05		-0.979	1	1		0.559
DPP6	1.18E-06		-1.004	0.907	0.964		0.016
SNCA	1.57E-08		-1.044	1	1		0.000
LOC103823960	1.61E-06		-1.078	0.953	1		0.022
GTF2H5	4.19E-07		-1.269	0.953	1		0.006
BCAS2	4.64E-07		-1.310	0.767	0.964		0.006
DDH36-mgp12	2.96E-10		-1.394	1	1		0.000
RPLP1	3.58E-06		-1.410	0.907	0.929		0.049
MRPS24	9.07E-06		-1.493	0.512	0.893		0.125
IGFBP7	3.25E-05		-1.493	0.558	0.893		0.448
LOC103817276	2.56E-05		-1.781	0.395	0.821		0.354
LOC127060513	5.77E-09		-1.866	0.628	1		0.000
HPGDS	1.05E-04		-2.114	0.233	0.643		1.000
SST	2.18E-05		-2.255	0.512	0.821		0.301
PENK	2.06E-05		-2.347	0.256	0.75		0.284
OST4	4.32E-11		-2.382	0.581	1		0.000
LOC108962641	6.00E-08		-2.789	0.116	0.75		0.001
LGALS1	8.54E-05		-2.841	0.047	0.429		1.000
CYP2J19	1.90E-05		-3.319	0.023	0.429		0.262
SLN	2.94E-05		-4.526	0	0.357		0.406
AR	3.11E-03		1.485	0.628	0.321		1.000
CCK	5.77E-03		-1.155	0.256	0.607		1.000
ESR1	7.22E-04		2.603	0.442	0.071		1.000

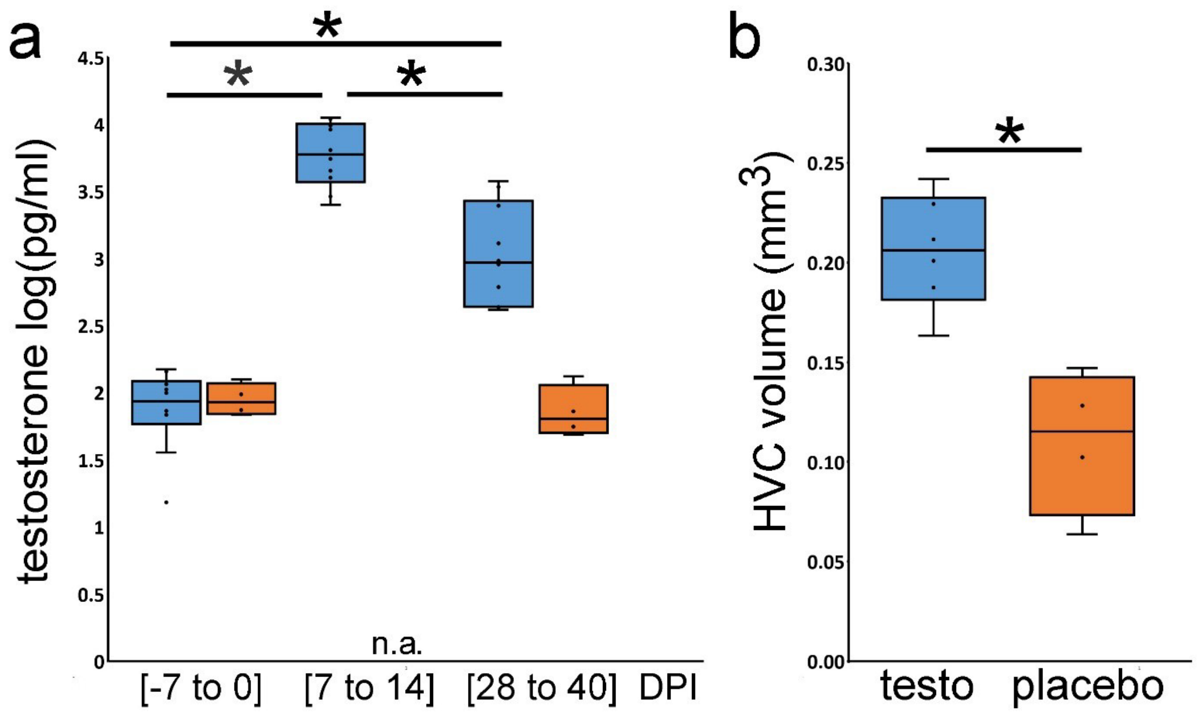

Supplementary Fig. S1. (**a**), In all testosterone-treated females (blue boxes), testosterone plasma levels were significantly higher 7 to 14 days after testosterone implantation (DPI), then dropped by DPI 28 to DPI 40, but were still significantly higher than in the week before treatment (DPI -7 to DPI 0) (one-way ANOVA of log transformed data, $f = 96.00$, $p < 0.00001$, Tukey HSD with $p < 0.00003$ for all comparisons). Testosterone levels were 88 ±43 pg/ml at DPI -7 to 0, 6680 ± 3312 pg/ml at DPI 7 to 14, and 1467 ± 1275 pg/ml at DPI 28 to 40. Testosterone levels in placebo-treated females (orange boxes) were similar before (DPI -7 to DPI 0: 91 ±28 pg/ml) and after (DPI 28 to 40: 77 ±38 pg/ml) implantation (one-sided t-test ($t(6) = 0.59$, $p = 0.287$). All testosterone data are expressed as mean ± sd. (**b**), the HVC volumes defined by cytoarchitecture (Nissl staining) were significantly larger in testosterone-treated (blue box) than in placebo-treated (orange box) females ($f(8) = 4.69$, $p = 0.0008$, one-tailed t-test). HVC volumes of the testosterone-treated females were 0.206 ± 0.028 mm^3 and were 0.110 ± 0.036 mm^3 of placebo-treated females; data are means ± sd. In (a) and (b), data are shown as boxplots, with dots representing inner data points and whiskers representing minimum and maximum data points. * = significant differences (see above). n.a. = not analyzed.

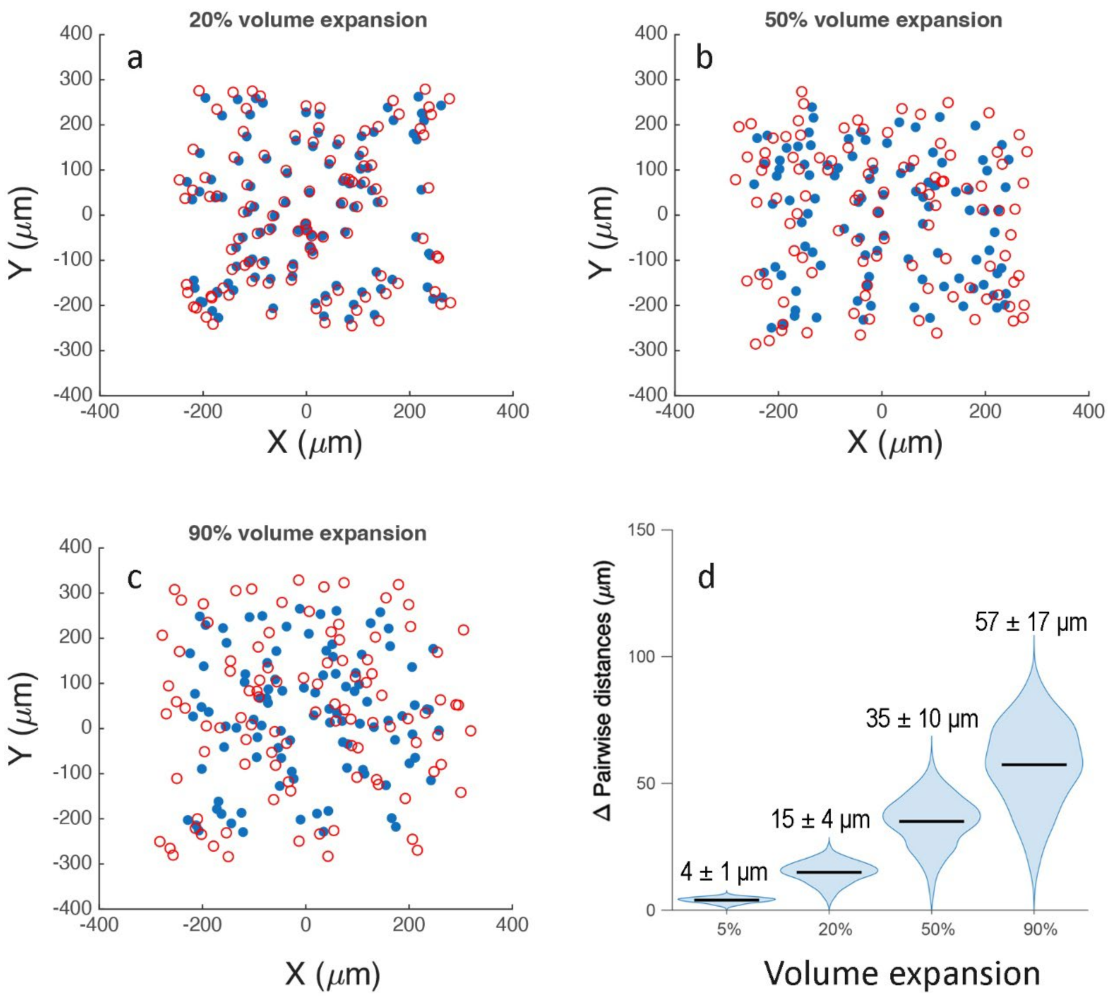

Supplementary Fig. S2: Estimation of cell displacements in connection with potential increases in HVC volume. HVC was modeled as a cube with a side length of 500 µm. Based on the resulting volume of 0.125 mm^3, we calculated the change in the pairwise distance between cells when the HVC volume increases uniform by 5%, 20% (**a**), 50% (**b**), and 90% (**c**). The latter corresponds to the average increase in HVC volume observed in the Nissl-stained sections of our testosterone-treated female canaries. In (a) to (c), the distribution of cells in the HVC at the start of expansion (blue) was randomized. The displaced cells are marked in red. In **d**, we present the modeling results of the changes in the pairwise cell distance. In the violin plot, the outlines were determined by the kernel density estimation of the probability density function. Solid lines mark the means, which are included as numbers, means ± standard deviations.

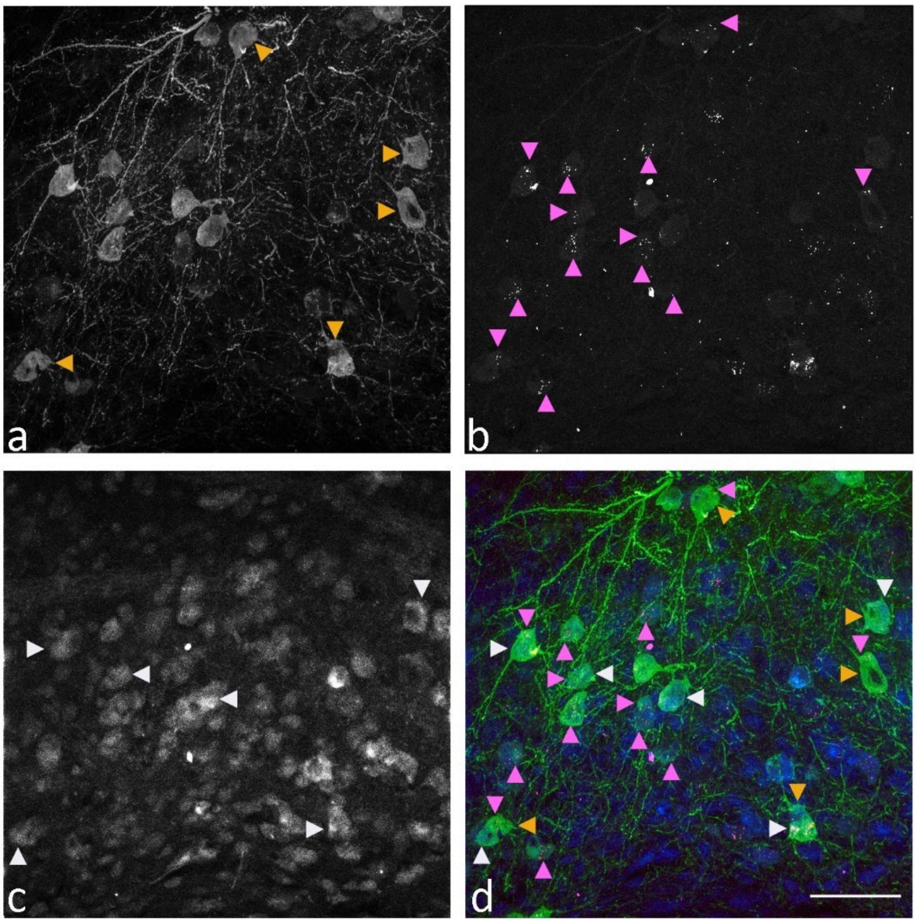

Supplementary Fig. S3: HVC neurons labelled by ssAAV9-CAG-GCaMP6s (GCaMP) include HVC_X projection neurons and GABAergic interneurons. In **a**, we show HVC neurons expressing GCaMP protein after local injection of AAV-GCaMP into HVC, immunocytochemically labeled with an GFP antibody. In **b**, HVC_X projection neurons (pink triangles) are labeled after injection of the retrograde tracer rhodamine-labeled Retrobeads into Area X. In **c**, GABAergic interneurons were labeled in an immunocytochemical staining for GAD65/67. Since (a) to (c) depict the same section, we superimposed these photomicrographs in (**d**): GAD-labeled cells are in blue, GFP-labeled cells are in green, and Retrobeads-labeled cells are with red dots. White triangles in (c) and (d) indicate some GFP-GAD double-labeled cells, with the green fluorescent label often obscuring the blue label. Orange triangles in (a) and (d) indicate some GFP-labeled cells that are neither GAD-labeled nor retrogradely-labeled. Likely, these cells are glutamatergic neurons of various kinds, including HVC_{RA} neurons. For pairwise distance measurements of cells, clusters were defined as one cell, i.e., no distance measurements between cells included in the same cluster were performed. Scale bar is 50 µm.

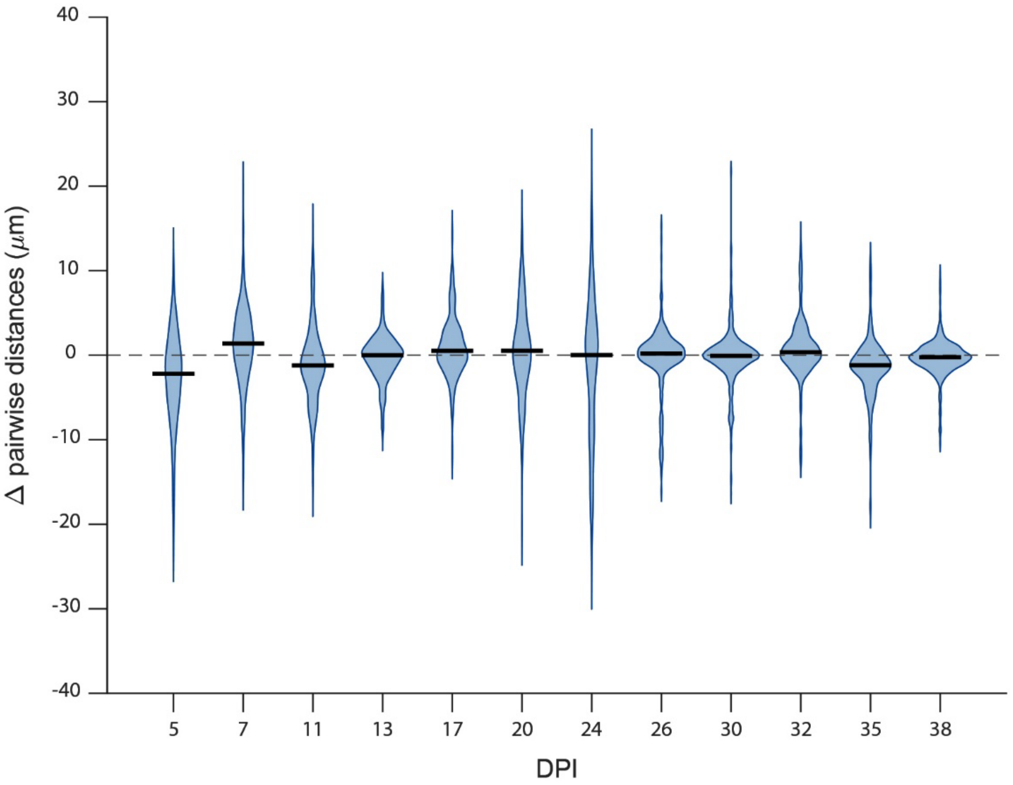

Supplementary Fig. S4: The distance between all types of HVC neurons labelled by ssAAV9CAG-GCaMP6s (GCaMP) is invariant during testosterone-induced song development in the female F18 (N = 54 cells). Viral injection labeled HVC neurons randomly, among which are HVC_X neurons and GABAergic interneurons (Supplementary Fig. S3). Depicted is the change in pairwise distance of labeled neurons between successive observation days throughout song development while full song was achieved between 32 and 35 days post implantation of testosterone (DPI). In the violin plot, the outlines were determined by the kernel density estimation of the probability density function. Solid black lines mark the medians, while the dashed grey line indicates the zero-reference, i.e., no change in pairwise distances between neurons. For statistics see Supplementary Tabs. S2: Comparisons of the observed pairwise-distances with expansion models showed significant differences of all observations for both the 10%-expansion and 5%-expansion models, and significant differences in 11 of 13 observation days with the 3%-expansion model (one-way ANOVAs [$f(1,25) = 86.6$, $p < 0.001$] followed by Tukey post hoc tests, Supplementary Tab. S2). Note that the small changes are both increases and decreases in pairwise distances, which would mean expansion and shrinkage (Supplementary Tab. S2).

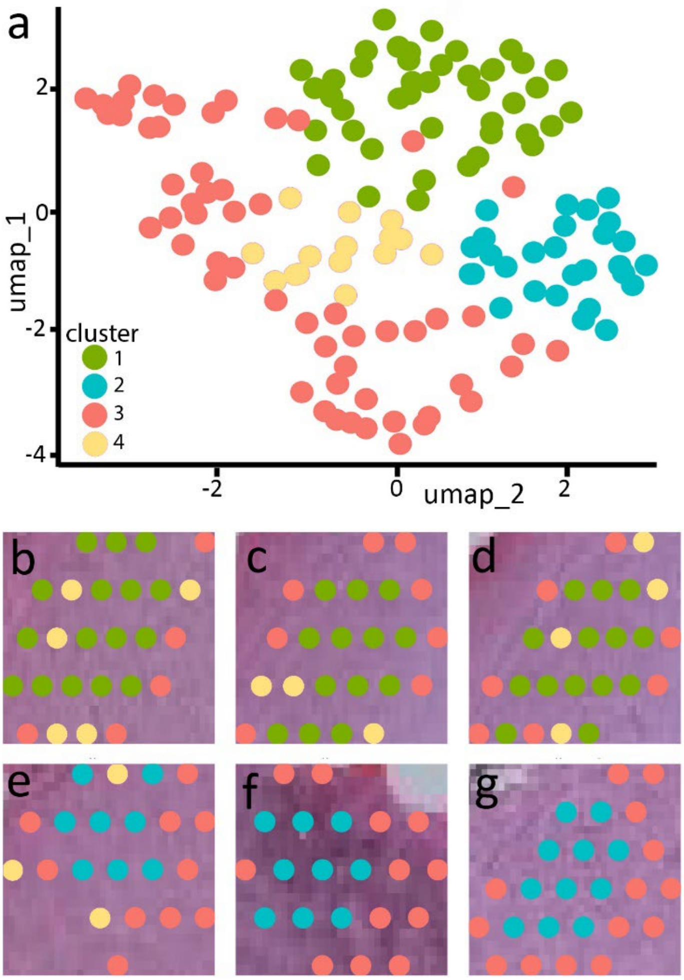

Supplementary Fig. S5. Testosterone increases the expression of a specific type of spatial transcriptomes in HVC. Combined analysis of the spatial transcriptomes (spots) located within the boundaries of HVC (defined by retrograde tracing) of testosterone-treated and placebo-treated canaries. (**a**) UMAP analysis of the 142 transcriptomes of HVC sections (b) to (g) resulted in four clusters: Cluster 1 (green spots; **b, c, d**) were located in the center of HVCs of testosterone-treated birds only and Cluster 2 (blue spots; **e, f, g**) were in the center of HVCs of placebo-treated birds only. Cluster 3 (red spots, b to g) are mainly located at the margins of retrogradely labelled HVCs and are reduced in number in testosterone-treated HVCs. Cluster 4 (yellow spots; b to g) is probably composed of mixtures of cells that otherwise form Clusters 1 and 3 or 2 and 3. Considered and depicted in (b) to (g) are only those spots, which were at least 50% located within the retrogradely-defined borders of HVC (see Fig. 3). The distribution of green and blue spots in (b) to (g) corresponds to the cytoarchitectonically-defined HVCs of testosterone-treated and placebo-treated birds, respectively (see Fig. 3). Note that the proportion of red transcriptomes among all HVC transcriptomes is a third in testosterone-treated birds and about two thirds in placebo-treated birds.

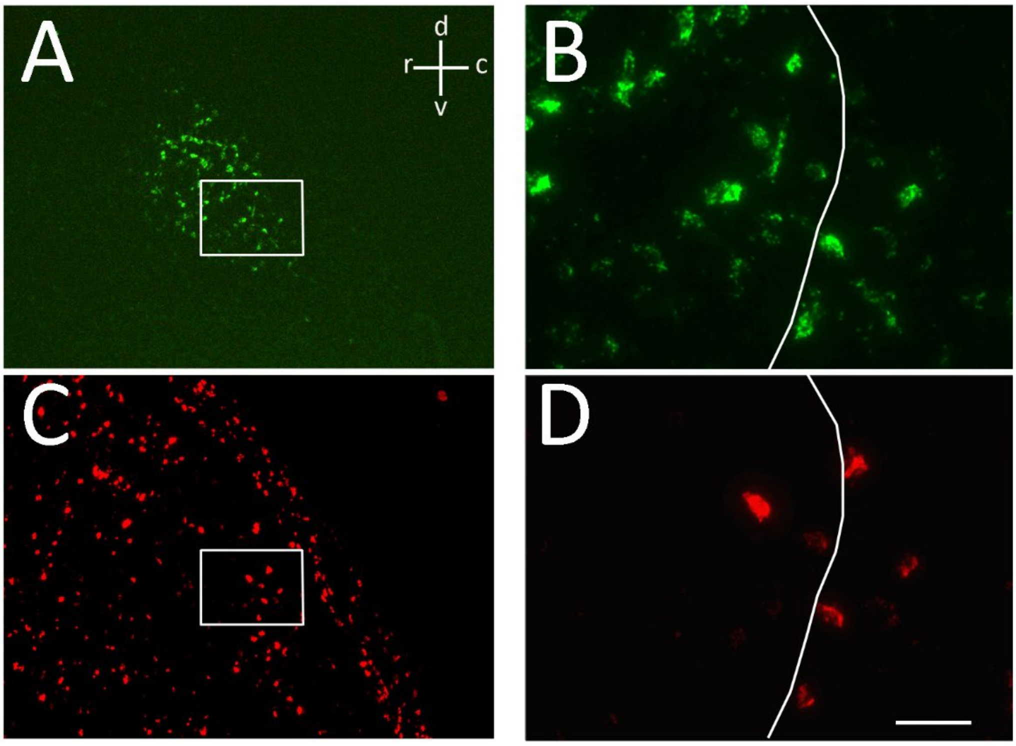

Supplementary Fig. S6: HVC areas underlying the spatial transcriptomes of Cluster 2 and Cluster 3 differ from each other in terms of gene expression. Differential gene expression of androgen receptor mRNA (AR mRNA, green labelled cells in A, B) and of cholecystokinin mRNA (CCK mRNA, red labelled cells in C, D) in the same HVC section of a placebo-treated female canary. Double-labeling is with RNAscope in-situ hybridizations. B and D show enlarged views of the boxed regions in A and C. The white lines (B, D) indicate the distribution of spots of Cluster 2 (left of white lines, HVC central) and Cluster 3 (right of while lines, HVC margin), as seen in a parallel section used for spatial transcriptomics. The distribution of AR mRNA expression indicates the anatomical boundaries of the HVC (A, B) and appears relatively uniform across its extent (see Tab. S4). In difference, the expression of CCK mRNA is lower in the central HVC compared to the HVC margin (see Supplementary Tab. S4). Further, CCK labelled cells are abundant in the nidopallium ventral (v) to HVC and in the hippocampus dorsal (d) to HVC. Dorsal (d) is at the top and caudal (c) is to the right in all panels. Scale bar represents 200 µm for A, C and 50 µm for B, D.

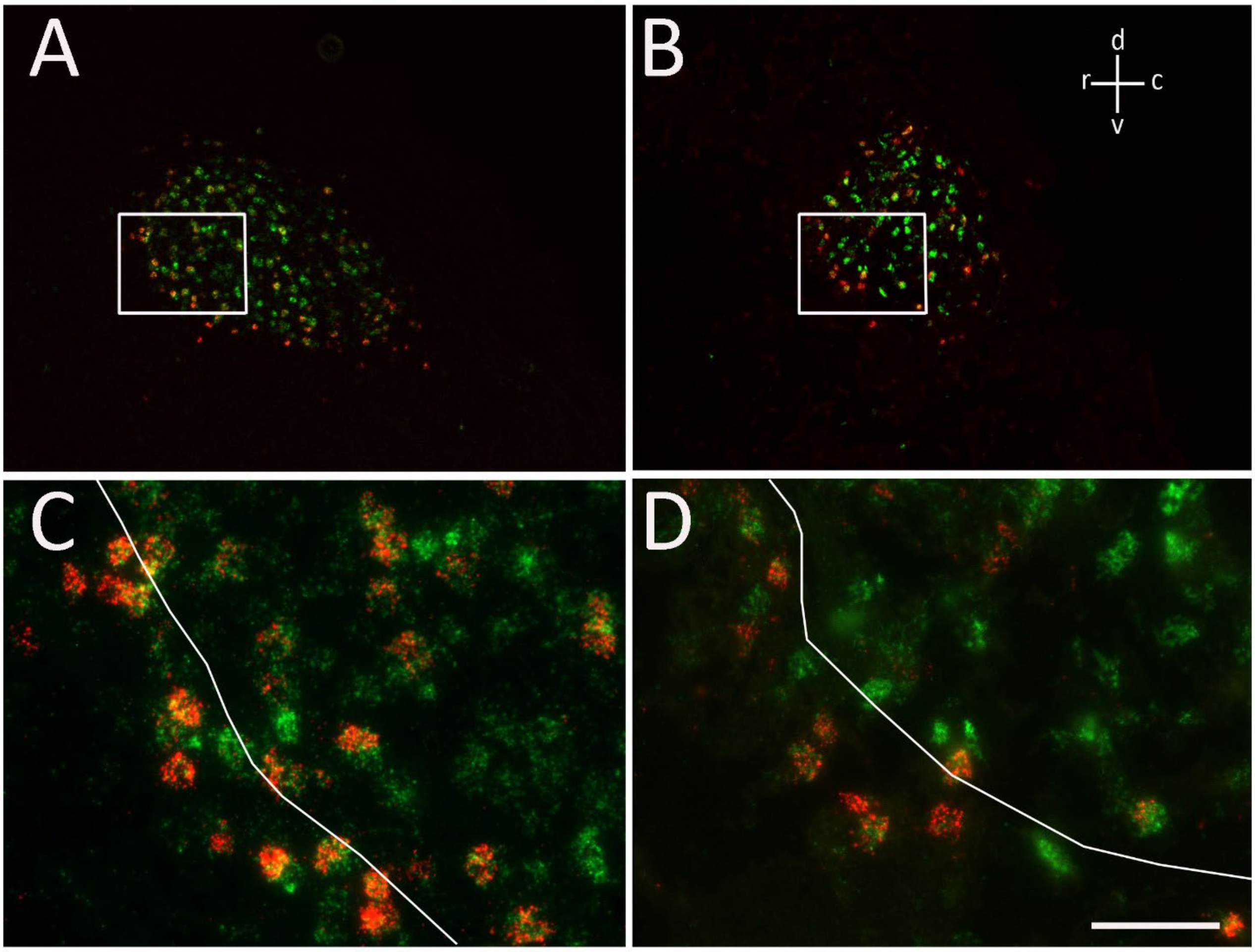

Supplementary Fig. S7: HVC sub-areas underlying the spatial transcriptomes of Cluster 1 and Cluster 2 differ from each other in terms of gene expression. Expression of estrogen receptor alpha (ESR1 mRNA, red labeled cells) is higher in the central HVC of a female canary treated with testosterone (**A**, **C**) compared to a placebo-treated female canary (**B**, **D**; see Supplementary Tab. S5). The distribution of androgen receptor (AR mRNA, green labeled cells) in the HVC appears relatively uniform, although slightly higher in the HVC of the testosterone-treated female, across the extent of the HVCs (A, B; see Supplementary Tab. S5). Double-labeling is with RNAscope in-situ hybridizations. C and D show enlarged views of the boxed regions in A and B. The white lines in C and D separate HVC sub-areas corresponding to the spatial transcriptome spots of the HVC center and the HVC margins, i.e., the areas to the left of the white line form the outer peripheral ring of spatial transcriptome spots seen in comparable sections (see Fig. 3, Supplementary Fig. S5). Areas to the right of the white lines are spatial transcriptomes of Cluster 1 in testosterone-treated females (C), and are spatial transcriptomes of Cluster 2 in placebo treated birds (D). Dorsal (d) is at the top and caudal (c) is to the right in all panels. Scale bar represents 200 μm for A, D, and 50 μm for C, D.

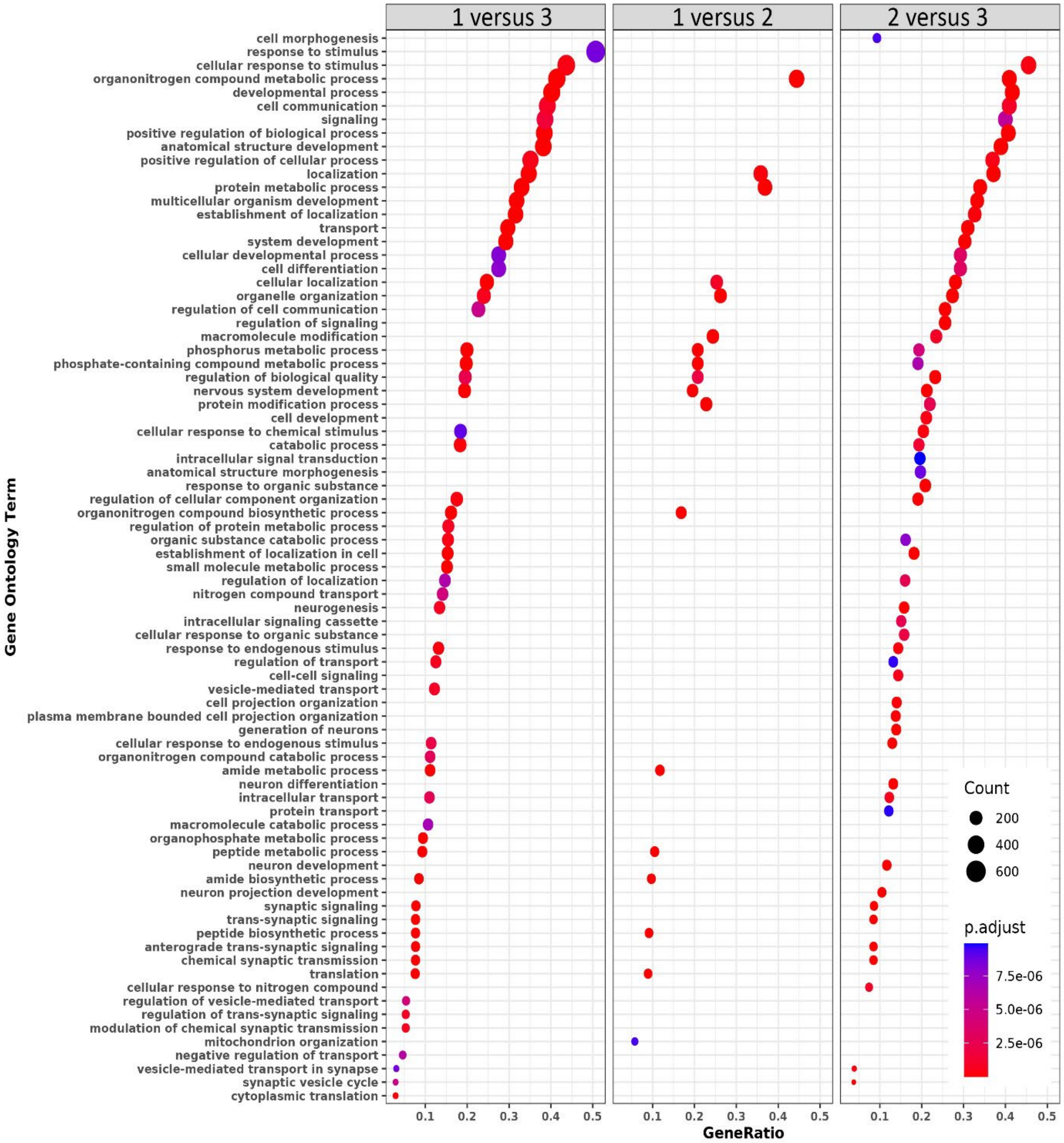

Supplementary Fig. S8. Gene ontology enrichment of Biological Processes (BP) of differentially expressed genes in spatial HVC transcriptomes of Cluster 1 versus Cluster 3, Cluster 1 versus Cluster 2, and Cluster 2 versus Cluster 3 (see Fig. 3 and Supplementary Fig. S3). Note that Cluster 1 is entirely in testosterone-treated birds and Cluster 2 in placebo-treated birds. Shown are BP categories (terms) with a significance level of $p < 0.00001$ (see Methods for statistical procedures). The Cluster 1 and 2 transcriptomes differ from those of Cluster 3 in many terms related to cell metabolism and differentiation, including various synaptic and axonal processes. In contrast, the

transcriptomes of Cluster 1 differ from those of Cluster 2 in far fewer processes, primarily related to metabolism and to a much lesser extent related to neuron differentiation, than in comparisons with Cluster 3. The sizes of the dots indicate the number of differentially expressed genes in the BP categories. GeneRatio is the ratio between the number of observed unique genes belonging to a Gene Ontology (GO) term and the total number of unique genes that make up that GO term.

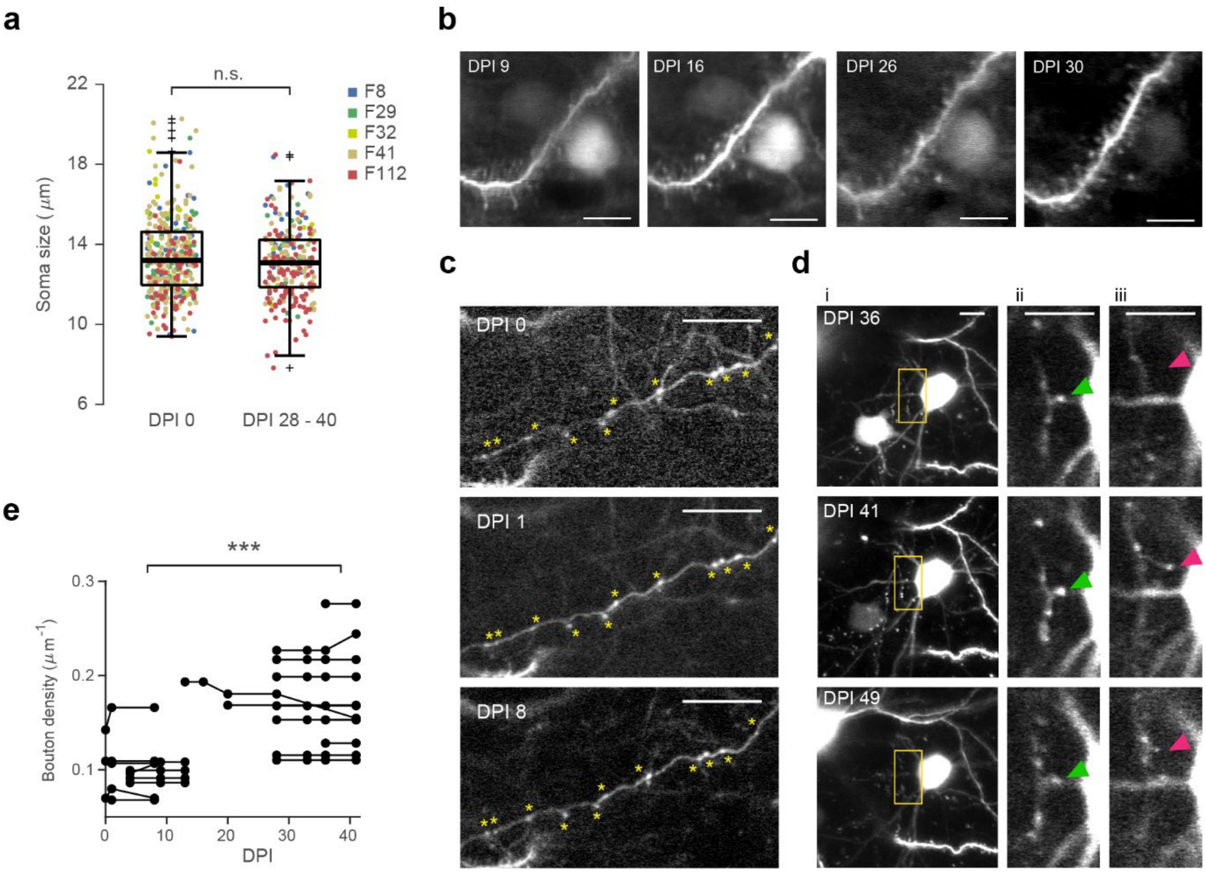

Supplementary Fig. S9: Testosterone treatment affects bouton densities but neither soma sizes nor dendritic spine densities of eGFP-labled HVC_X neurons during song development. DPI = days post testosterone implantation (**a**) Soma sizes at DPI 0 (no song) and at DPI 28 - 40 (full song) are similar, indicating stability in soma sizes during song development. Each dot is one data point, color-coded for the five testosterone-treated females. (**b**) Dendritic spine distributions 2-photon-imaged across DPI 9, DPI 18, DPI 26 and DPI 30 showed no notable changes, as depicted for one dendrite. In difference, 2-photon imaging revealed stable bouton configuration during early song development (**c**) but dynamic boutons during full song stage (**d**). **(dii)** and **(diii)** are zoomed views of (**di**) (yellow windows) at two different focal depths, respectively. Highlighted are a persistent bouton (green arrows, dii) and remodeling of boutons (magenta arrows, diii). (**e**) Bouton densities during full song stage (DPI 28 to DPI 42) are significantly higher compared to early stages of song development (DPI 0 to DPI 10). n.s. = not significant; *** = $p < 0.001$. Scale bars are 10 µm in b and d (i, ii, iii), and 20 µm in c.

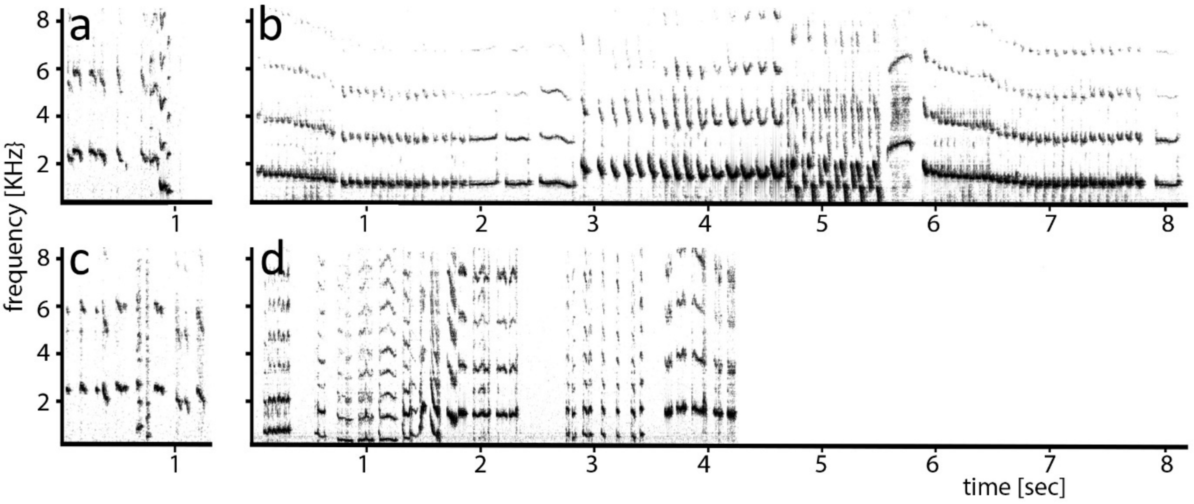

Supplementary Fig. S10: Testosterone-induced singing in seven-year-old female canaries F23 (**a, b**) and F24 (**c, d**). Females started to sing 6 (a) respectively 3 (c) days after testosterone implantation (DPI). At DPI 16, the song of F23 was already very stable (b) while that of F24 was not yet fully developed.
